# Supplementary material for: Comparing severe COVID-19 outcomes of first and second/third waves: a prospective single-centre cohort study of health-related quality of life and pulmonary outcomes 6 months after infection
Source: BMJ Open. 2023 Jul 17;13(7):e071394. doi: 10.1136/bmjopen-2022-071394 (PMC10357304; doi:10.1136/bmjopen-2022-071394)
Supplement: Supplementary data [file bmjopen-2022-071394supp001.pdf]

SUPPLEMENTARY FILE

Supplementary Table 1. Comparisons of patient characteristics between participants and non-participants among those invited for follow-up.

Characteristics	Participants	Non-participants	p-values	Missing
Total number, n (%)	278 (85)	50 (15)		
Age, years mean±SD	59.5± 11.9	62.0± 13.7	0.18	
Sex, n (%)<br>Male	203 (73)	37 (74)	0.56	
SAPS 3 score, mean±SD	55.6± 7.8	55.8± 7.6	0.90	51
Ventilator treatment, n (%)<br>Invasive	136 (49)	15 (30)	<0.05	

Student's t-test and Chi_2-test were used where appropriate. Statistically significant p-value <0.05.

Supplementary Table 2. Comparisons of patient characteristics between participants and non-participants including all survivors >3 months.

Characteristics	Participants	Non-participants	p-values	Missing
Total number, n (%)	278 (61)	178 (39)		
Age, years mean±SD	59.5± 11.9	61.1± 13.4	0.19	
Sex, n (%)<br>Male	203 (73)	128 (72)	0.83	
SAPS 3 score, mean±SD	55.6± 7.8	55.7± 7.6	0.95	82
Ventilator treatment, n (%)<br>Invasive	136 (49)	89 (50)	0.85	

Student's t-test and Chi_2-test were used where appropriate. Statistically significant p-value <0.05.

Supplementary Table 3. Comparison between participants who completed the questionnaires; performed the pulmonary function test; chest CT scan and those who did not.

Measure	Characteristic	Responder	Non-responder	p-value
**RAND-36**		N=187	N=91	
	Age ≥ 65	66 (36)	27 (29)	0.35
	Male	134 (72)	69 (76)	0.46
	Diabetes	39 (21)	21 (23)	0.67
	Hypertension/ cardiovascular disease	99 (53)	40 (44)	0.16
	Chronic lung disease	43 (23)	16 (18)	0.30
	Mechanical ventilation	91 (49)	45 (50)	0.90
	Corticosteroid treatment	143 (76)	62 (68)	0.14
**Pulmonary function test**		N=206	N=72	
	Age ≥ 65	73 (35)	20 (28)	0.24
	Male	149 (72)	54 (75)	0.67
	Diabetes	41 (20)	19 (26)	0.25
	Hypertension/ cardiovascular disease	103 (50)	36 (50)	1.00
	Chronic lung disease	44 (21)	15 (21)	0.92
	Mechanical ventilation	104 (50)	32 (44)	0.38
	Corticosteroid treatment	148 (72)	57 (79)	0.22
**Chest CT scan**		N=212	N=66	
	Age ≥ 65	71 (34)	22 (33)	0.98
	Male	151 (71)	52 (79)	0.23
	Diabetes	46 (22)	14 (21)	0.93
	Hypertension/ cardiovascular disease	104 (49)	35 (53)	0.57
	Chronic lung disease	49 (23)	10 (15)	0.17
	Mechanical ventilation	107 (50)	29 (44)	0.35
	Corticosteroid treatment	155 (73)	50 (76)	0.67

Supplementary Table 4. Univariate analysis of patient- and clinical characteristics in relation to health-related quality of life (HRQL) in COVID-19 intensive care unit survivors presented as adjusted mean score differences (MSD) with 95% confidence intervals (CI).

**Variables**	Number	**Physical function** MSD (95%CI) p-value	**Role physical** MSD (95%CI) p-value	**Bodily pain** MSD (95%CI) p-value	**General health** MSD (95%CI) p-value	**Vitality** MSD (95%CI) p-value	**Social function** MSD (95%CI) p-value	**Role emotional** MSD (95%CI) p-value	**Mental health** MSD (95%CI) p-value
**Age, years**									
≤65	120	Reference 1.0	Reference 1.0	Reference 1.0	Reference 1.0	Reference 1.0	Reference 1.0	Reference 1.0	Reference 1.0
>65	66	-13.3* (-21.3 to -5.3) <0.01	1.0 (-12.5 to 14.4) 0.89	-5.2 (-14.1 to 3.6) 0.24	-1.1 (-7.9 to 5.6) 0.74	3.3 (-4.2 to 10.8) 0.38	-1.7 (-10.4 to 7.0) 0.70	3.4 (-9.6 to 16.3) 0.61	2.5 (-3.7 to 8.8) 0.42
**Sex**									
Female	53	Reference 1.0	Reference 1.0	Reference 1.0	Reference 1.0	Reference 1.0	Reference 1.0	Reference 1.0	Reference 1.0
Male	133	6.5 (-2.2 to 15.2) 0.14	13.8 (-0.3 to 27.9) 0.06	4.6 (-4.7 to 14.0) 0.33	5.6 (-1.6 to 12.7) 0.12	8.6* (0.7-16.4) 0.03	10.2* (1.1 to 19.2) 0.03	1.8 (-11.9 to 15.5) 0.80	1.8 (-4.8 to 8.4) 0.60
**Diabetes**									
No	147	Reference 1.0	Reference 1.0	Reference 1.0	Reference 1.0	Reference 1.0	Reference 1.0	Reference 1.0	Reference 1.0
Yes	39	-11.5* (-21.0 to -2.0) 0.02	-6.74 (-22.5 to 9.0) 0.4	-10.2 (-20.5 to 0.1) 0.05	-10.0* (-17.8 to -2.2) 0.01	-4.5 (-13.3 to 4.3) 0.31	-3.1 (-13.3 to 7.1) 0.55	-10.1 (-25.2 to 5.1) 0.19	-8.3* (-15.6 to -1.1) 0.02
**Hypertension/ cardiovascular disease**									
No	87	Reference 1.0	Reference 1.0	Reference 1.0	Reference 1.0	Reference 1.0	Reference 1.0	Reference 1.0	Reference 1.0
Yes	99	-7.9* (-15.7 to -0.7) 0.05	0.5 (-12.4 to 13.4) 0.94	-7.4 (-15.8 to 1.0) 0.08	0.0 (-6.5 to 6.5) 1.0	2.3 (-4.9 to 9.5) 0.52	1.2 (-7.1 to 9.5) 0.78	0.8 (-11.6 to 13.2) 0.90	1.95 (-4.1 to 7.9) 0.52
**Chronic lung disease**									
No	144	Reference 1.0	Reference 1.0	Reference 1.0	Reference 1.0	Reference 1.0	Reference 1.0	Reference 1.0	Reference 1.0
Yes	42	-7.0 (-16.3 to 2.4) 0.14	-15.1 (-30.2 to -0.0) 0.05	-5.4 (-15.5 to 4.6) 0.29	-7.2 (-14.8 to 0.4) 0.06	-9.7* (-18.1 to -1.3) 0.02	-6.0 (-15.8 to 3.9) 0.23	-20.5* (-34.8 to -6.1) <0.01	-2.6 (-9.7 to 4.4) 0.46

**Ventilation support**									
HFNO/NIV	95	Reference 1.0	Reference 1.0	Reference 1.0	Reference 1.0	Reference 1.0	Reference 1.0	Reference 1.0	Reference 1.0
invasive ventilation	91	-5.6 (-13.4 to 2.3) 0.16	-12.4 (-25.1 to 0.4) 0.06	-12.3* (-20.6 to -4.0) <0.01	-2.2 (-8.6 to 4.3) 0.5	-1.4 (-8.6 to 5.8) 0.70	-7.8 (-16.0 to 0.5) 0.06	-1.26 (-13.7 to 11.2) 0.84	2.8 (-3.2 to 8.8) 0.36
**In-ICU corticosteroids**									
No	43	Reference 1.0	Reference 1.0	Reference 1.0	Reference 1.0	Reference 1.0	Reference 1.0	Reference 1.0	Reference 1.0
Yes	143	-3.6 (-12.9 to 5.7) 0.44	5.7 (-9.4 to 20.8) 0.46	3.3 (-6.6 to 13.3) 0.5	1.9 (-5.7 to 9.5) 0.6	3.6 (-4.8 to 12.0) 0.40	2.6 (-7.2 to 12.4) 0.60	9.1 (-5.4 to 23.6) 0.22	6.4 (-0.6 to 13.3) 0.07

Abbreviations: HFNO=high flow nasal oxygen; NIV=non-invasive ventilation. *Statistically significant p-value <0.05.
