# Supplementary material for: Comparing severe COVID-19 outcomes of first and second/third waves: a prospective single-centre cohort study of health-related quality of life and pulmonary outcomes 6 months after infection
Source: BMJ Open. 2023 Jul 17;13(7):e071394. doi: 10.1136/bmjopen-2022-071394 (PMC10357304; doi:10.1136/bmjopen-2022-071394)
Supplement: Author's manuscript [file bmjopen-2022-071394.draft_revisions.pdf]

BMJ Open is committed to open peer review. As part of this commitment we make the peer review history of every article we publish publicly available.

When an article is published we post the peer reviewers' comments and the authors' responses online. We also post the versions of the paper that were used during peer review. These are the versions that the peer review comments apply to.

The versions of the paper that follow are the versions that were submitted during the peer review process. They are not the versions of record or the final published versions. They should not be cited or distributed as the published version of this manuscript.

BMJ Open is an open access journal and the full, final, typeset and author-corrected version of record of the manuscript is available on our site with no access controls, subscription charges or pay-per-view fees (http://bmjopen.bmj.com).

If you have any questions on BMJ Open's open peer review process please email info.bmjopen@bmj.com

BMJ Open

Comparing severe COVID-19 outcomes of first and second/third waves: a prospective single-centre cohort study of health-related quality of life and pulmonary outcomes 6 months after infection

Journal:	*BMJ Open*
Manuscript ID	bmjopen-2022-071394
Article Type:	Original research
Date Submitted by the Author:	26-Dec-2022

Complete List of Authors:	Darlington, Pernilla; Karolinska Institute Department of Clinical Science and Education Södersjukhuset, Department of Internal Medicine; Karolinska Institutet, Department of Clinical Science and Education<br>Roël , Mari; Karolinska Institute Department of Clinical Science and Education Södersjukhuset, Department of Internal Medicine<br>Cronhjort , Maria; Karolinska Institutet, Department of Clinical Science and Education<br>Hanna, Gabriel; Karolinska Institute Department of Clinical Science and Education Södersjukhuset<br>Hedman , Anders; Karolinska Institutet, Department of Clinical Science and Education<br>Joelsson-Alm , Eva; Karolinska Institutet, Department of Clinical Science and Education<br>Schandl, Anna; Karolinska Institutet, Department of Clinical Science and Education

Keywords:	COVID-19, Quality of Life, Chest imaging < RADIOLOGY & IMAGING

SCHOLARONE™
Manuscripts

I, the Submitting Author has the right to grant and does grant on behalf of all authors of the Work (as defined in the below author licence), an exclusive licence and/or a non-exclusive licence for contributions from authors who are: i) UK Crown employees; ii) where BMJ has agreed a CC-BY licence shall apply, and/or iii) in accordance with the terms applicable for US Federal Government officers or employees acting as part of their official duties; on a worldwide, perpetual, irrevocable, royalty-free basis to BMJ Publishing Group Ltd ("BMJ") its licensees and where the relevant Journal is co-owned by BMJ to the co-owners of the Journal, to publish the Work in this journal and any other BMJ products and to exploit all rights, as set out in our licence.

The Submitting Author accepts and understands that any supply made under these terms is made by BMJ to the Submitting Author unless you are acting as an employee on behalf of your employer or a postgraduate student of an affiliated institution which is paying any applicable article publishing charge ("APC") for Open Access articles. Where the Submitting Author wishes to make the Work available on an Open Access basis (and intends to pay the relevant APC), the terms of reuse of such Open Access shall be governed by a Creative Commons licence – details of these licences and which Creative Commons licence will apply to this Work are set out in our licence referred to above.

Other than as permitted in any relevant BMJ Author's Self Archiving Policies, I confirm this Work has not been accepted for publication elsewhere, is not being considered for publication elsewhere and does not duplicate material already published. I confirm all authors consent to publication of this Work and authorise the granting of this licence.

For peer review only

2022-12-26

Comparing severe COVID-19 outcomes of first and second/third waves: a prospective single-centre cohort study of health-related quality of life and pulmonary outcomes 6 months after infection

Authors: Pernilla DARLINGTON[1,2]*, Mari ROËL[1]*, Maria CRONHJORT[2,3], Gabriel HANNA[4], Anders HEDMAN[2,5], Eva JOELSSON-ALM[2,3], Anna SCHANDL[2,3].

*Shared first authorship

Affiliations: [1]Department of Internal Medicine, Södersjukhuset, Stockholm; [2]Department of Clinical Science and Education, Södersjukhuset, Karolinska Institutet, Sweden; [3]Department of Anesthesia and intensive care, Södersjukhuset Stockholm, Sweden; [4]Department of Radiology, Södersjukhuset, Stockholm, Sweden; [5]Department of Cardiology, Södersjukhuset Stockholm, Sweden.

Corresponding author: Pernilla Darlington, Department of Internal Medicine, Södersjukhuset, Stockholm, Sjukhusbacken 10, 118 83. Stockholm, e-mail address: pernilla.darlington@ki.se

Keywords: COVID-19, CT, quality of life, chest imaging

For peer review only - http://bmjopen.bmj.com/site/about/guidelines.xhtml

2022-12-26

ABSTRACT

Objective

The clarify whether outcomes in intensive care unit (ICU) survivors treated for severe COVID-19 infection have improved with time.

Design

Prospective cohort study.

Setting

Single-centre study of adult COVID-19 patients with respiratory distress admitted to two Swedish ICUs during the first wave (1 March–1 September 2020) and second/third waves (2 September 2020– 1 August 2021).

Participants

456 critically ill COVID-19 patients who survived for at least 90 days.

Main outcome measures

Outcomes were differences in health-related quality of life (HRQL) and pulmonary function between waves. General linear regression and multivariable logistic regression were used to present mean score differences (MSD) and odds ratios (OR) with 95% confidence intervals (CI).

Results

Among the 456 (67%) critically ill COVID-19 patients who survived for at least 90 days after ICU discharge, 278 (61%) attended follow-up approximately 6 months after ICU discharge. HRQL was similar between the groups, except that the second/third wave-survivors scored

For peer review only - http://bmjopen.bmj.com/site/about/guidelines.xhtml

2022-12-26

higher in the role physical domain (MSD 26.7, 95%CI: 8.8-44.8) and lower on bodily pain (MSD 14.8, 95%CI: 3.0-26.5). Patients in the second/third waves had lower diffusion capacity for carbon monoxide (DLCO) (OR 2.1, 95%CI: 1.2-3.6), but after adjustment for age, sex, tobacco smoking, chronic lung disease, invasive ventilation and corticosteroid treatment, this association no longer remained statistically significant (OR 1.5, 95%CI: 0.7-3.3).

Conclusions

This study indicates that even though intensive care treatment strategies have improved with time, long-term HRQL and impaired respiratory health remain similar at 6 months for patients surviving severe COVID-19 in the first and second/third waves of the pandemic.

Strength and limitations of this study

*The main methodological strengths of the study lie in the prospective and comprehensive data collection of long-term outcomes in critically ill patients with COVID-19 together with the use of well-validated outcome tests and questionnaires.

*Limitation of the study is the risk of selection bias as some COVID-19 survivors opted out of the follow-up program. Though, when comparing baseline data for all survivors, no statistically significant differences were found.

*Since data on virus stainstrains were not available, we could not adjust for this parameter and potential confounding effects cannot be ruled out.

For peer review only - http://bmjopen.bmj.com/site/about/guidelines.xhtml

2022-12-26

INTRODUCTION

Coronavirus disease 2019 (COVID-19), caused by severe acute respiratory syndrome coronavirus 2 (SARS-CoV-2), was declared a global pandemic in March 2020 and has resulted in numerous severely ill individuals in need of respiratory support[1] and millions of deaths worldwide[2]. The majority survived the acute episode of a critical illness but were confronted with physical and psychological sequelae after discharge from hospital which prolonged recovery[3]. Approximately 35-80% of the survivors experienced persistent symptoms of fatigue, dyspnea, joint pain, sleep disorders, and loss of memory and concentration lasting beyond 4 weeks after a moderate to severe acute infection, which is referred to as "post-acute COVID-19 syndrome"[4-6] Long-lasting respiratory complications as well as other functional deficits which may cause substantial morbidity have been reported among intensive care unit (ICU) survivors in the first year after hospital discharge[7-10]. Furthermore, a high number of residual abnormalities on chest CT-scan have been found several months after discharge from hospital, and acute respiratory distress syndrome has been associated with more severe radiological findings[11]. Since the start of the pandemic the risk for hospital admission has varied as new variants of SARS-CoV-2 have emerged [12]. Likewise, worldwide different virus variants have been dominating the waves in Sweden [13]. Further, during the second and third waves, scientific and clinical understanding of the illness improved, and potentially more effective treatments were provided. Yet, whether functional outcomes have improved in line with the increase in knowledge about COVID-19 remains unexplored. Therefore, this prospective single-centre cohort study aimed to clarify whether outcomes in ICU survivors treated for severe COVID-19 infection have improved with time and to identify factors associated with worse long-term outcomes.

For peer review only - http://bmjopen.bmj.com/site/about/guidelines.xhtml

METHODS

Study design and setting

This was a prospective, single-centre cohort study including all patients with acute COVID-19 infection who were admitted to two ICUs at Södersjukhuset, Stockholm, Sweden between 1 March 2020 and 1 August 2021. In Sweden, the first cases of severely ill patients were admitted to the ICU at the beginning of March 2020 and this first wave lasted until August – with a few cases during the summer. Therefore, this time period has been considered to separate the end of the first wave. The beginning of the second wave was estimated to start at 2 September 2020. There was no clear gap in time between the second and third waves, but there was a moderate decrease in March 2021, and the second and third waves were therefore grouped as one time period in this study. Thus, the study was categorized into two time periods: the first wave (1 March–1 September 2020) and the second/third waves (2 September 2020–1 August 2021). The main differences in treatment strategies between the two time periods were that corticosteroids became standard treatment in the ICU at the end of the first wave. Also, there was a shift from early invasive ventilation to allowing non-invasive ventilation during the second/third waves.

Ethical considerations

Study approval was granted from the Swedish Ethical Review Authority (Diarie number: 2020-03760) and written informed consent was obtained from all included participants.

For peer review only - http://bmjopen.bmj.com/site/about/guidelines.xhtml

2022-12-26

Participants

All critically ill patients with a positive polymerase chain reaction test for COVID-19 and who were treated for respiratory failure with invasive ventilation, high-flow treatment with oxygen (HFNO) or non-invasive ventilation (NIV) in the ICU were eligible for inclusion in the study. Surviving patients were invited for a follow-up in the hospital at six months after hospital discharge. Together with the invitation for follow-up, a questionnaire assessing health-related quality of life was sent to the patients. At follow-up the patients met a nurse and a physician for recapitulation of their ICU stay. The patients were invited on separate occasions for pulmonary function testing and a chest CT-scan.

Data Collection

Data on demographic and clinical details were recorded from medical charts regarding age, sex, body mass index (BMI), smoking habits, comorbidities, Simplified Acute Physiological Score III (SAPS 3; a scoring system used to predict mortality risk in the ICU, where a higher score indicates a higher mortality risk [14]), corticosteroid treatment, respiratory support (invasive/NIV/HFNO) and ICU length of stay.

Outcomes

The outcomes were health-related quality of life (HRQL) and pulmonary impairments on chest CT-scan, and capacity for gas-exchange as a measurement of pulmonary function approximately 6 months after ICU discharge between the first and second/third waves.

For peer review only - http://bmjopen.bmj.com/site/about/guidelines.xhtml

2022-12-26

HRQL was assessed with the 36-item questionnaire RAND-36 Item Health Survey (RAND Corp). The questionnaire includes the following eight domains: physical functioning (10 items), role physical (4 items), bodily pain (2 items), general health (5 items), vitality (4 items), social functioning (2 items), role emotional (3 items) and emotional well-being (5 items)[15]. Questionnaire responses were linearly transformed into scores between 0 and 100, where a higher score represents a higher HRQL[15, 16]. Minimally clinically important differences were set to 3-5 point differences between groups[17]. Missing items were accounted for with automatic calculation of the scores. If more than half of the scores in one domain were missing, no result was presented.

Pulmonary impairments were identified with a CT scan of the thoracic region using a Siemens Somatom Drive with a standard 120-kV CT thorax protocol as described in a previous publication[7] . International standard thoracic radiological terminology from the Fleischner Society[18] was used to identify the parenchymal changes related to SARS-CoV-2 infection,[19] such as ground-glass opacities, subpleural bands, reticular pattern, and bronchiectasis. A semi-quantitative CT score (CT involvement score) was estimated based on lobar involvement and then calculated as a total score for all five lobes: 0: 0%, 1: <5%, 2: 5%–25%, 3: 26%–50%, 4: 51%–75%, 5: >75%, yielding a score ranging from 0 to 5 in each lobe and a global score of 0–25[20].

The capacity for gas exchange, i.e. diffusion capacity for carbon monoxide (DLCO) (uncorrected value), was measured according to standard methods[21]. DLCO values were expressed in accordance with reference values (Hedenström)[22, 23] with DLCO <80% as cut-off for impaired pulmonary function[8, 9, 11, 24].

For peer review only - http://bmjopen.bmj.com/site/about/guidelines.xhtml

2022-12-26

Statistical analyses

Multivariable general linear regression models were used to estimate mean score differences (MSD) with 95% CI between groups for RAND-36 scores and multivariable logistic regression models were used to assess lung function changes between first and second/third waves and are presented as odds ratios (OR) with 95% confidence intervals (CI). The models were adjusted for age (≤50, 50-65, >65 years), sex (male/female), comorbidity (diabetes, hypertension, cardiovascular disease, and chronic lung disease), tobacco smoking habits (ever/never), corticosteroids (yes/no) and invasive ventilation (yes/no). Two separate statistical analyses were conducted including patients with at least one test result, and patients who had complete data on the three examinations (chest CT-scan, pulmonary function test and questionnaires). Statistical analyses were performed with Graph Pad Prism 8 (Jamovi version 2.3.19) and an experienced biostatistician validated all analyses.

Patient and public involvement

Patients and the public were not involved in the development of this study.

For peer review only - http://bmjopen.bmj.com/site/about/guidelines.xhtml

2022-12-26

RESULTS

Participants

In total, 678 patients were treated for severe COVID-19 infection during the study period, among which 456 (67%) survived for at least 90 days after ICU discharge. Of these, 278 (61%) attended the follow-up evaluation approximately 6 months after ICU discharge (Figure 1). First-wave survivors were younger, had lower BMI, fewer cardiovascular diseases, higher CRP max, and were more often treated with invasive ventilation, but less frequently with corticosteroids (Table 1). Among survivors with chronic lung disease, the vast majority had asthma. Patient characteristics and ICU-related data were similar between survivors who attended the follow-up and non-participants (Supplementary Table 1), but among non-responders who declined follow-up, non-invasive ventilation was more common (Supplementary Table 2).

For peer review only

For peer review only - http://bmjopen.bmj.com/site/about/guidelines.xhtml

2022-12-26

Table 1. Characteristics of included COVID-19 intensive care unit survivors across the first and second/third pandemic waves

	**First wave** (1 March–1 Sept 2020)	**Second/third waves** (2 Sept 2020 – 1 Aug 2021)	**p-value**
Total number, n	122	156	
Follow-up time, months, Md (IQR)	5 (4-5)	6 (5-8)	<0.001*
Age, years Md (IQR)	58 (50-66)	61 (53-69)	<0.05*
Male n (%)	93 (76)	110 (71)	0.34
BMI, kg/m^2 Md (IQR)	28 (25-31)	29 (26-33)	<0.05*
Diabetes n (%)	24 (20)	36 (23)	0.56
Hypertension n (%)	53 (43)	72 (46)	0.72
Cardiovascular disease n (%)	12 (10)	30 (19)	<0.05*
Chronic lung disease n (%)	22 (18)	37 (24)	0.30
Ever smoker n (%)	46 (38)	74 (47)	0.11
CRP max, mg/L, Md (IQR)	310 (199-355)	182 (126-248)	<0.001*
SAPS 3, Md (IQR)	54 (50-60)	56 (52-59)	0.52
Invasive ventilation n (%)	72 (59)	64 (41)	<0.01*
Corticosteroid treatment n (%)	49 (40)	156 (100)	<0.001*
Length of ICU stay (days), Md (IQR)	13 (4-23)	9 (6-16)	0.33

BMI= body mass index; CRP = C reactive protein; IQR=Interquartile range; Md=Median; SAPS= Simplified Acute Physiology Score (missing information in 37 participants); *p value <0.05

HRQL

Among the 187 ICU survivors who reported HRQL data, 75 were treated in the ICU for COVID-19 during the first wave. First-wave survivors reported lower scores in all HRQL domains, with clinically relevant and statistically significant differences in the role physical domain (MSD 26.7, 95%CI: 8.8 to 44.8), bodily pain (MSD 14.8, 95%CI: 3.0 to 26.5) and general health (MSD 10.3, 95%CI: 1.3 to 19.3) (Table 2). Similar results were found for

For peer review only - http://bmjopen.bmj.com/site/about/guidelines.xhtml

2022-12-26

survivors of COVID-19 with complete case data, except for non-significant results for general health (MSD 10.8, 95%CI: -0.4 to 22.1).

Table 2. Health-related quality of life (HRQL) comparing COVID-19 intensive care unit survivors across the first and second/third waves, presented as mean score differences (MSD) with 95% confidence intervals (CI).

Health-related quality of life domains	**First wave**	**Second/third waves**	**Unadjusted analysis** Mean score differences (95%CI)	**Adjusted analysis** Mean score differences (95%CI)
	Mean scores (95%CI)	Mean scores (95%CI)		
Total number	75	111		
Physical function	62 (57-68)	64 (58-69)	1.3 (-6.7 to 9.3)	7.3 (-3.5 to 18.0)
Role physical	31 (22-40)	50 (41-58)	18.5* (5.6 to 31.3)	26.7* (8.8-44.8)
Bodily pain	59 (52-66)	69 (64-75)	10.8* (2.3 to 19.3)	14.8* (3.0 to 26.5)
General health	49 (44-54)	56 (51-60)	6.3 (-0.2 to 12.8)	10.3* (1.3 to 19.3)
Vitality	47 (41-52)	53 (48-58)	6.3 (-0.9 to 13.5)	8.3 (-1.8 to 18.3)
Social function	62 (56-68)	67 (62-73)	5.6 (-2.9 to 14.0)	5.8 (-5.9 to 17.5)
Role emotional	58 (48-68)	68 (60-75)	9.9 (-2.6 to 22.5)	10.8 (-6.8 to 28.4)
Mental health	67 (62-72)	74 (70-78)	6.8* (0.8 to 12.8)	7.0 (-1.5 to 15.6)

The model was adjusted for age, sex, tobacco smoking, chronic lung disease, corticosteroid treatment and invasive ventilation. *Statistically significant p-value <0.05.

In the multivariate analysis, older age (MSD -12.7, 95%CI: -21.0 to -4.5) was associated with worse physical function. Survivors with hypertension/cardiovascular disease were more likely to report lower scores on bodily pain (MSD -9.0, 95%CI: -17.6 to -0.5) as were those with invasive ventilation (MSD -10.9, 95%CI: -19.3 to -2.6) (Table 3). In the complete case analysis, results for invasive ventilation no longer remained significant. However, chronic

For peer review only - http://bmjopen.bmj.com/site/about/guidelines.xhtml

2022-12-26

lung disease was associated with lower scores in the role emotional domain (MSD -34.3, 95%CI: -54.4 to -14.2). The univariate analyses can be found in Supplementary Table 3.

Table 3. Multivariate analysis of patient- and clinical characteristics in relation to health- related quality of life (HRQL) in COVID-19 intensive care unit survivors presented as adjusted mean score differences (MSD) with 95% confidence intervals (CI).

**Variables**	Number	**Physical function** MSD (95%CI)	**Role physical** MSD (95%CI)	**Bodily pain** MSD (95%CI)	**General health** MSD (95%CI)	**Vitality** MSD (95%CI)	**Social function** MSD (95%CI)	**Role emotional** MSD (95%CI)	**Mental health** MSD (95%CI)
**Age, years**									
≤65	120	Reference 1.0	Reference 1.0	Reference 1.0	Reference 1.0	Reference 1.0	Reference 1.0	Reference 1.0	Reference 1.0
>65	66	-12.7* (-21.0 to -4.5)	-1.3 (-14.8 to 12.3)	-5.1 (-13.9 to 3.7)	-1.9 (-8.9 to 5.0)	2.4 (-5.2 to 10.1)	-2.5 (-11.5 to 6.4)	1.7 (-11.6 to 15.0)	1.7 (-4.7 to 8.2)
**Sex**									
Female	53	Reference 1.0	Reference 1.0	Reference 1.0	Reference 1.0	Reference 1.0	Reference 1.0	Reference 1.0	Reference 1.0
Male	133	5.1 (-3.8 to 14.1)	10.6 (-4.3 to 25.3)	4.3 (-5.3 to 13.8)	3.6 (-3.9 to 11.2)	6.3 (-2.0 to 14.7)	9.5 (-0.2 to 19.2)	-4.8 (-19.2 to 9.6)	1.0 (-6.1 to 8.0)
**Hypertension/ cardiovascular disease**									
No	87	Reference 1.0	Reference 1.0	Reference 1.0	Reference 1.0	Reference 1.0	Reference 1.0	Reference 1.0	Reference 1.0
Yes	99	-6.3 (-14.3 to 1.7)	-3.6 (-16.7 to 9.6)	-9.0* (-17.6 to -0.5)	-0.9 (-7.7 to 5.8)	-0.1 (-7.6 to 7.4)	-0.5 (-9.2 to 8.2)	-2.2 (-15.1 to 10.8)	0.6 (-5.7 to 6.9)
**Chronic lung disease**									
No	144	Reference 1.0	Reference 1.0	Reference 1.0	Reference 1.0	Reference 1.0	Reference 1.0	Reference 1.0	Reference 1.0
Yes	42	-5.8 (-15.6 to 4.0)	-13.6 (-29.4 to 2.2)	-5.6 (-15.9 to 4.7)	-6.9 (-15.0 to 1.2)	-8.3 (-17.2 to 0.7)	-3.0 (-13.4 to 7.5)	-24.1* (-39.6 to -8.7)	-3.5 (-11.1 to 4.1)
**Ventilation support**									
HFNO/NIV	95	Reference 1.0	Reference 1.0	Reference 1.0	Reference 1.0	Reference 1.0	Reference 1.0	Reference 1.0	Reference 1.0
invasive ventilation	91	-5.6 (-13.5 to 2.2)	-7.6 (-20.6 to 5.4)	-10.9* (-19.3 to -2.6)	-0.5 (-7.1 to 6.1)	0.3 (-7.0 to 7.6)	-7.0 (-15.6 to 1.5)	1.5 (-11.2 to 14.2)	4.2 (-2.0 to 10.3)
**In-ICU corticosteroids**									
No	43	Reference 1.0	Reference 1.0	Reference 1.0	Reference 1.0	Reference 1.0	Reference 1.0	Reference 1.0	Reference 1.0
Yes	143	-6.4 (-18.8 to 5.9)	-12.4 (-32.8 to 8.1)	-4.8 (-18.1 to 8.5)	-5.0 (-15.5 to 5.4)	-1.9 (-13.5 to 9.7)	-0.8 (-14.3 to 12.7)	3.2 (-16.8 to 23.1)	1.0 (-8.8 to 10.7)

Abbreviations: HFNO=high flow nasal oxygen; NIV=non-invasive ventilation; All the variables in the univariate analysis were included in the multivariate analysis with adjustment for time during the pandemic. *Statistically significant p-value <0.05.

Pulmonary outcomes

Compared with first wave-survivors, patients in the second/third waves had lower DLCO, indicating worse pulmonary function and more residual changes on chest CT-scan, even though the latter was non-significant. However, in the adjusted analysis, pulmonary

For peer review only - http://bmjopen.bmj.com/site/about/guidelines.xhtml

2022-12-26

outcomes were similar between groups- (Table 4). Similar non-significant findings were seen in patients who had complete data from the three examinations.

Table 4. Odds ratios (OR) with 95% confidence intervals (CI) of CT-score and diffusion capacity (DLCO) comparing COVID-19 intensive care unit survivors across the first and second/third pandemic waves. First wave patients' data were used as a reference.

Outcomes	**First wave**		**Second/third waves**		**Unadjusted model** OR (95%CI)	**Adjusted model** OR (95%CI)
	Numbers	Median (IQR)	Numbers	Median (IQR)		
CT-score						
0	25	0	21	0	Reference 1.0	Reference 1.0
≥1	65	7 (5-10)	101	9 (5-13)	1.8 (1.0-3.6)	0.7 (0.2-2.2)
DLCO % of predicted						
≥80	64	96 (90-109)	49	91 (86-96)	Reference 1.0	Reference 1.0
<80	36	68 (62-75)	57	65 (53-71)	2.1 (1.2-3.6)*	1.5 (0.7-3.3)

Abbreviations: DLCO=diffusion capacity for carbon monoxide; CT=computed tomography; IQR = interquartile range. The models were adjusted for age, sex, tobacco smoking, chronic lung disease, corticosteroid treatment and invasive ventilation. *p-value <0.05.

Factors associated with worse radiological pulmonary outcome (i.e. CT-score≥1) were older age (>65 years) (OR 4.5, CI: 1.7-11.7), male sex (OR 2.8, CI: 1.2-6.2) and ICU corticosteroid treatment (OR 4.6, CI: 1.3-16.4) (Table 5). For the complete case analysis, only older age was associated with worse CT-scores (OR 3.1, 95%CI: 1.0 to 9.5).

Worse pulmonary function (DLCO<80%) was associated with older age (OR 1.9, CI: 1.0-3.5) and female sex (with female as reference) (OR 0.5, CI: 0.2-0.9) (Table 5), but in the complete case analysis only female sex was associated with worse capacity for gas-exchange (OR 0.3, 95%CI: 0.1 to 0.9).

For peer review only - http://bmjopen.bmj.com/site/about/guidelines.xhtml

Table 5. Patient- and clinical characteristics in relation to pulmonary outcomes in COVID-19 intensive care unit survivors presented as odds ratio (OR) with 95% confidence intervals (CI).

Variables	CT-score≥1			DLCO <80%		
	Numbers	OR (95%CI) Univariate	OR (95%CI) Multivariate	Numbers	OR (95%CI) Univariate	OR (95%CI) Multivariate
**Age, years**						
≤65	141	Reference 1.0	Reference 1.0	133	Reference 1.0	Reference 1.0
>65	71	4.3 (1.8-10.7)*	4.5 (1.7-11.7)*	73	2.2 (1.2-3.9)*	1.9 (1.0-3.5)*
**Sex**						
Female	61	Reference 1.0	Reference 1.0	57	Reference 1.0	Reference 1.0
Male	151	1.8 (0.9-3.6)	2.8 (1.2-6.2)*	149	0.5 (0.3-0.9)*	0.5 (0.2-0.9)*
**Hypertension/ cardiovascular disease**						
No	108	Reference 1.0	Reference 1.0	103	Reference 1.0	Reference 1.0
Yes	104	1.4 (0.7-2.7)	1.1 (0.5-2.3)	103	1.8 (1.0-3.2)*	1.7 (0.9-3.2)
**Chronic lung disease**				162	Reference 1.0	Reference 1.0
No	163	Reference 1.0	Reference 1.0	44	1.1 (0.6-2.2)	0.8 (0.4-1.8)
Yes	49	1.1 (0.5-2.4)	1.6 (0.7-4.0)			
**Ventilation support**						
HFNO/NIV	105	Reference 1.0	Reference 1.0	102	Reference 1.0	Reference 1.0
invasive ventilation	107	1.4 (0.7-2.8)	1.4 (0.7-3.0)	104	1.2 (0.7-2.0)	1.3 (0.7-2.5)
**In-ICU corticosteroids**						
No	57	Reference 1.0	Reference 1.0	58	Reference 1.0	Reference 1.0
Yes	155	3.0 (1.5-6.0)*	4.6 (1.3-16.4)*	148	2.5 (1.3-4.9)*	1.7 (0.7-4.1)

Abbreviations: DLCO=diffusion capacity for carbon monoxide; HFNO=high flow nasal oxygen; NIV=non-invasive ventilation; multivariate analysis included all the variables in the univariate analysis and was adjusted for time during the pandemic. *Statistically significant p-value <0.05.

For peer review only

For peer review only - http://bmjopen.bmj.com/site/about/guidelines.xhtml

DISCUSSION

In this prospective cohort study of COVID-19 ICU survivors, HRQL and pulmonary outcomes were relatively similar between the pandemic waves, despite a changing patient case-mix, new virus variants and improved intensive care treatment strategies, except that second/third wave-survivors scored higher in the role physical domain and had less bodily pain.

Most evidence indicates a small reduction in mortality rates between the first and second/third pandemic waves among critically ill patients[25, 26]. However, data about mortality trends during the pandemic vary depending on study time frames, outcomes, case-mix and sample size[2]. Only a few studies have investigated whether long-term morbidity follows the mortality results. One of these studies was a multinational study, in which 1000 patients with COVID-19 and severe hypoxaemia were randomized to either 6 mg or 12 mg dexamethasone, but no differences between groups were found in 3-months mortality or HRQL[27].

A systematic review and meta-analysis showed that those with more severe initial illness have poorer long-term recovery after COVID-19 infection[28]. Since the COVID-19 treatment strategies have improved with time, we hypothesized that patients treated later in the pandemic would have less post-ICU morbidity, which was also found for role physical and general health. However, for pulmonary function no such improvement was observed. For pulmonary healing, older age and corticosteroid treatment were associated with more residual changes on chest CT-scan. Age as a risk factor for severe disease and worse radiological outcome is well-known and has been described previously[9, 29]. So far, few studies have investigated the long-term effects of corticosteroid treatment for pulmonary healing in critically ill patients with COVID-19. Corticosteroid treatment has been used to

For peer review only - http://bmjopen.bmj.com/site/about/guidelines.xhtml

reduce the inflammation seen in COVID-19 patients. However, recent evidence has shown that even if there may be short-term mortality benefits for mechanically ventilated patients, there may also be negative long-term mortality consequences[30]. A randomized controlled trial showed that corticosteroid treatment improved forced vital capacity in hospitalized patients with COVID-19, but diffusion capacity, DLCO, was not included in the study[31]. Another study showed that the rate of ventilator-associated pneumonia was higher during the latter part of the pandemic, when the use of corticosteroid treatment had also increased, suggesting different causes of final respiratory failure between the COVID-19 waves[25].

Increasing age, female sex and hypertension/cardiovascular disease were associated with worse lung function at follow-up. In general, there was a predominance of males who become critically ill[32]. The females in need of ICU treatment in our cohort may have had a worse pulmonary impairment before COVID-19 because there was a discrepancy between CT-score and the capacity for gas-exchange (DLCO) in females. An association between female sex and worse DLCO values in hospitalized patients has also been reported by others[24].

Strength and limitations

The main methodological strengths of the study lie in the prospective and comprehensive data collection together with the use of well-validated outcome tests and questionnaires. The study also has a number of limitations. Firstly, there is the risk of selection bias as some COVID-19 survivors opted out of the follow-up program. Of those who declined follow-up,

For peer review only - http://bmjopen.bmj.com/site/about/guidelines.xhtml

fewer had needed invasive ventilation and they might have reached complete recovery and considered follow-up as unnecessary. Though, when comparing baseline data for all survivors, no statistically significant differences were found. Furthermore, during the second/third waves, 51 survivors were excluded from the study because of reasons we do not know. During this time, the hospital was under heavy burden of severely ill patients and all ICU nurses and physicians were needed in bedside work and the follow-up had to be done retrospectively. However, these data were most likely missed at random. Also, the lack of baseline data prevents identification of new-onset problems. Yet, data on pulmonary function and HRQL is difficult to obtain pre-ICU. The HRQL results were self-reported and may include a risk of response shift. Also, the time interval for follow-up varied which might have had an impact on the results. However, mean time to follow-up was similar between the comparison groups. As HRQL aspects include many variables, and several analyses were conducted, there may be a risk that some statistically significant results may be due to chance. Lastly, results were adjusted for some known confounders. However, since data on virus strains were not available, we could not adjust for this parameter and potential confounding effects cannot be ruled out.

This cohort included a heterogenic COVID-19 population who received care at a general hospital in the Stockholm area. We assume that in countries where healthcare organization is similar, the results of this study are likely to be generalizable and may contribute to knowledge on how to assess and accommodate expected problems after severe COVID-19 disease and respiratory distress. Given the increasing cumulative number of patients who survive after severe COVID-19 infection, it is important to understand long-term functional outcomes in order to clarify patients' rehabilitation needs and inform development of evidence-based interventions which will help prevent new disabilities. Further research is

For peer review only - http://bmjopen.bmj.com/site/about/guidelines.xhtml

For peer review only

needed to improve survivorship in ICU patients with COVID-19 infection, and it is of great importance to investigate what factors may negatively affect recovery.

CONCLUSION

This study suggests that even though patient case-mix has changed, new variants of SARS-CoV-2 have emerged and intensive care treatment strategies have improved since the start of the pandemic, long-term HRQL and impaired respiratory health remains similar for COVID-19 patients with critical illness.

For peer review only

For peer review only - http://bmjopen.bmj.com/site/about/guidelines.xhtml

DECLARATIONS

Ethics approval and consent to participate

Study approval was granted from the Swedish Ethical Review Authority (Diarie number: 2020-03760) and written informed consent was obtained from all included participants.

Declaration of interests

The authors declare that they have no competing interests.

Availability of data and materials

The datasets used and/or analysed during the current study are available from the corresponding author upon reasonable request.

Funding

Departmental funding only.

Author contributions

PD and MR contributed to conceptualisation, methodology, formal analysis, investigation, writing – original draft. MC contributed to methodology, investigation, writing – review and editing. GH contributed to methodology, investigation, writing – review and editing. AH: conceptualisation, investigation, writing – review and editing. EJA contributed to conceptualisation, formal analysis, writing – review and editing. AS contributed to conceptualisation, methodology, investigation, formal analysis, writing draft – review and editing. All authors read and approved the final manuscript.

For peer review only - http://bmjopen.bmj.com/site/about/guidelines.xhtml

For peer review only

Acknowledgements

We would like to thank all patients who participated in the study and caregivers involved in the follow-up.

For peer review only

For peer review only - http://bmjopen.bmj.com/site/about/guidelines.xhtml

REFERENCES

1. Lim ZJ, Subramaniam A, Ponnapa Reddy M, et al. Case Fatality Rates for Patients with COVID-19 Requiring Invasive Mechanical Ventilation. A Meta-analysis. *Am J Respir Crit Care Med*. 2021; 203: 54-66.
2. Fan G, Yang Z, Lin Q, Zhao S, Yang L and He D. Decreased Case Fatality Rate of COVID-19 in the Second Wave: A study in 53 countries or regions. *Transbound Emerg Dis*. 2021; 68: 213-5.
3. Evans RA, McAuley H, Harrison EM, et al. Physical, cognitive, and mental health impacts of COVID-19 after hospitalisation (PHOSP-COVID): a UK multicentre, prospective cohort study. *Lancet Respir Med*. 2021; 9: 1275-87.
4. Chopra V, Flanders SA, O'Malley M, Malani AN and Prescott HC. Sixty-Day Outcomes Among Patients Hospitalized With COVID-19. *Ann Intern Med*. 2021; 174: 576-8.
5. Carfi A, Bernabei R, Landi F and Gemelli Against C-P-ACSG. Persistent Symptoms in Patients After Acute COVID-19. *JAMA*. 2020; 324: 603-5.
6. Garrigues E, Janvier P, Kherabi Y, et al. Post-discharge persistent symptoms and health-related quality of life after hospitalization for COVID-19. *J Infect*. 2020; 81: e4-e6.
7. Hanna G, Bankler S, Schandl A, et al. The role of ventilatory support for long-term outcomes after critical infection with COVID-19: A prospective cohort study. *Clin Respir J*. 2022; 16: 63-71.
8. Schandl A, Hedman A, Lynga P, et al. Long-term consequences in critically ill COVID-19 patients: A prospective cohort study. *Acta Anaesthesiol Scand*. 2021; 65: 1285-92.
9. Huang C, Huang L, Wang Y, et al. 6-month consequences of COVID-19 in patients discharged from hospital: a cohort study. *Lancet*. 2021; 397: 220-32.
10. Jacobson KB, Rao M, Bonilla H, et al. Patients With Uncomplicated Coronavirus Disease 2019 (COVID-19) Have Long-Term Persistent Symptoms and Functional Impairment Similar to Patients with Severe COVID-19: A Cautionary Tale During a Global Pandemic. *Clin Infect Dis*. 2021; 73: e826-e9.
11. Han X, Fan Y, Alwalid O, et al. Six-month Follow-up Chest CT Findings after Severe COVID-19 Pneumonia. *Radiology*. 2021; 299: E177-E86.
12. Twohig KA, Nyberg T, Zaidi A, et al. Hospital admission and emergency care attendance risk for SARS-CoV-2 delta (B.1.617.2) compared with alpha (B.1.1.7) variants of concern: a cohort study. *Lancet Infect Dis*. 2022; 22: 35-42.
13. Strålin K, Bruce D, Wahlström E, et al. Impact of the Alpha VOC on disease severity in SARS-CoV-2-positive adults in Sweden. *J Infect*. 2022; 84: e3-e5.
14. Metnitz PG, Moreno RP, Almeida E, et al. SAPS 3--From evaluation of the patient to evaluation of the intensive care unit. Part 1: Objectives, methods and cohort description. *Intensive Care Med*. 2005; 31: 1336-44.
15. Hays RD, Sherbourne CD and Mazel RM. The RAND 36-Item Health Survey 1.0. *Health Econ*. 1993; 2: 217-27.
16. Orwelius L, Nilsson M, Nilsson E, et al. The Swedish RAND-36 Health Survey - reliability and responsiveness assessed in patient populations using Svensson's method for paired ordinal data. *J Patient Rep Outcomes*. 2017; 2: 4.
17. Samsa G, Edelman D, Rothman ML, Williams GR, Lipscomb J and Matchar D. Determining clinically important differences in health status measures: a general approach with illustration to the Health Utilities Index Mark II. *Pharmacoeconomics*. 1999; 15: 141-55.
18. Hansell DM, Bankier AA, MacMahon H, McLoud TC, Muller NL and Remy J. Fleischner Society: glossary of terms for thoracic imaging. *Radiology*. 2008; 246: 697-722.
19. Chung M, Bernheim A, Mei X, et al. CT Imaging Features of 2019 Novel Coronavirus (2019-nCoV). *Radiology*. 2020; 295: 202-7.
20. Francone M, Iafrate F, Masci GM, et al. Chest CT score in COVID-19 patients: correlation with disease severity and short-term prognosis. *Eur Radiol*. 2020; 30: 6808-17.
21. Graham BL, Brusasco V, Burgos F, et al. 2017 ERS/ATS standards for single-breath carbon monoxide uptake in the lung. *Eur Respir J*. 2017; 49.

For peer review only - http://bmjopen.bmj.com/site/about/guidelines.xhtml

22. Hedenstrom H, Malmberg P and Fridriksson HV. Reference values for lung function tests in men: regression equations with smoking variables. *Ups J Med Sci*. 1986; 91: 299-310.
23. Hedenstrom H, Malmberg P and Agarwal K. Reference values for lung function tests in females. Regression equations with smoking variables. *Bull Eur Physiopathol Respir*. 1985; 21: 551-7.
24. Mendez R, Latorre A, Gonzalez-Jimenez P, et al. Reduced Diffusion Capacity in COVID-19 Survivors. *Ann Am Thorac Soc*. 2021; 18: 1253-5.
25. Carbonell R, Urgeles S, Rodriguez A, et al. Mortality comparison between the first and second/third waves among 3,795 critical COVID-19 patients with pneumonia admitted to the ICU: A multicentre retrospective cohort study. *Lancet Reg Health Eur*. 2021; 11: 100243.
26. Dongelmans DA, Termorshuizen F, Brinkman S, et al. Characteristics and outcome of COVID-19 patients admitted to the ICU: a nationwide cohort study on the comparison between the first and the consecutive upsurges of the second wave of the COVID-19 pandemic in the Netherlands. *Ann Intensive Care*. 2022; 12: 5.
27. Granholm A, Kjaer MN, Munch MW, et al. Long-term outcomes of dexamethasone 12 mg versus 6 mg in patients with COVID-19 and severe hypoxaemia. *Intensive Care Med*. 2022; 48: 580-9.
28. Han Q, Zheng B, Daines L and Sheikh A. Long-Term Sequelae of COVID-19: A Systematic Review and Meta-Analysis of One-Year Follow-Up Studies on Post-COVID Symptoms. *Pathogens*. 2022; 11.
29. Chen Y, Ding C, Yu L, et al. One-year follow-up of chest CT findings in patients after SARS-CoV-2 infection. *BMC Med*. 2021; 19: 191.
30. Moreno G, Carbonell R, Martin-Loeches I, et al. Corticosteroid treatment and mortality in mechanically ventilated COVID-19-associated acute respiratory distress syndrome (ARDS) patients: a multicentre cohort study. *Ann Intensive Care*. 2021; 11: 159.
31. Barros C, Freire RS, Frota E, et al. Short-Course of Methylprednisolone Improves Respiratory Functional Parameters After 120 Days in Hospitalized COVID-19 Patients (Metcovid Trial): A Randomized Clinical Trial. *Front Med (Lausanne)*. 2021; 8: 758405.
32. Peckham H, de Gruijter NM, Raine C, et al. Male sex identified by global COVID-19 meta-analysis as a risk factor for death and ITU admission. *Nat Commun*. 2020; 11: 6317.

For peer review only

For peer review only - http://bmjopen.bmj.com/site/about/guidelines.xhtml

Figure legend

Figure 1. Flowchart of study inclusion.

For peer review only

For peer review only - http://bmjopen.bmj.com/site/about/guidelines.xhtml

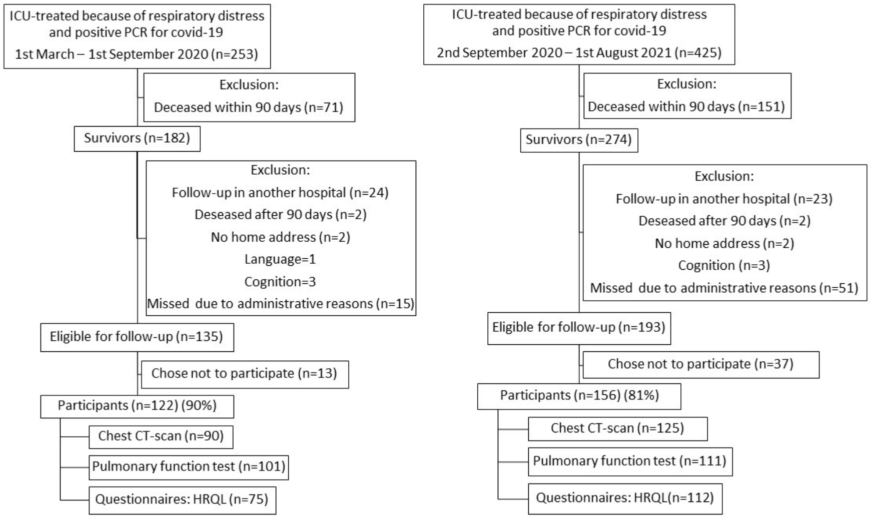

Flowchart of study inclusion.

338x190mm (96 x 96 DPI)

SUPPLEMENTARY FILE

Supplementary Table 1. Comparisons of patient characteristics between participants and non-participants among those invited for follow-up

Characteristics	Participants	Non-participants	p-values	Missing
Total number, n (%)	278 (85)	50 (15)		
Age, years mean±SD	59.5± 11.9	62.0± 13.7	0.18	
Sex, n (%)<br>Male	203 (73)	37 (74)	0.56	
SAPS 3 score, mean±SD	55.6± 7.8	55.8± 7.6	0.90	51
Ventilator treatment, n (%)<br>Invasive	136 (49)	15 (30)	<0.05	

Comparisons between groups were performed with Student's t-test and Chi_2-test where appropriate. Statistically significant p-value <0.05.

SUPPLEMENTARY FILE

Supplementary Table 2. Comparisons of patient characteristics between participants and non-participants including all survivors >3 months.

Characteristics	Participants	Non-participants	p-values	Missing
Total number, n (%)	278 (61)	178 (39)		
Age, years mean±SD	59.5± 11.9	61.1± 13.4	0.19	
Sex, n (%)<br>Male	203 (73)	128 (72)	0.83	
SAPS 3 score, mean±SD	55.6± 7.8	55.7± 7.6	0.95	82
Ventilator treatment, n (%)<br>Invasive	136 (49)	89 (50)	0.85	

Comparisons between groups were performed with Student's t-test and Chi_2-test where appropriate. Statistically significant p-value <0.05.

For peer review only

Supplementary Table 3. Univariate analysis of patient- and clinical characteristics in relation to health-related quality of life (HRQL) in COVID-19 intensive care unit survivors presented as adjusted mean score differences (MSD) with 95% confidence intervals (CI).

Variables	Number	**Physical function** MSD (95%CI)	**Role physical** MSD (95%CI)	**Bodily pain** MSD (95%CI)	**General health** MSD (95%CI)	**Vitality** MSD (95%CI)	**Social function** MSD (95%CI)	**Role emotional** MSD (95%CI)	**Mental health** MSD (95%CI)
**Age, years**									
≤65	120	Reference 1.0	Reference 1.0	Reference 1.0	Reference 1.0	Reference 1.0	Reference 1.0	Reference 1.0	Reference 1.0
>65	66	-13.3* (-21.3 to -5.3)	1.0 (-12.5 to 14.4)	-5.2 (-14.1 to 3.6)	-1.1 (-7.9 to 5.6)	3.3 (-4.2 to 10.8)	-1.7 (-10.4 to 7.0)	3.4 (-9.6 to 16.3)	2.5 (-3.7 to 8.8)
**Sex**									
Female	53	Reference 1.0	Reference 1.0	Reference 1.0	Reference 1.0	Reference 1.0	Reference 1.0	Reference 1.0	Reference 1.0
Male	133	6.5 (-2.2 to 15.2)	13.8 (-0.3 to 27.9)	4.6 (-4.7 to 14.0)	5.6 (-1.6 to 12.7)	8.6* (0.7-16.4)	10.2* (1.1 to 19.2)	1.8 (-11.9 to 15.5)	1.8 (-4.8 to 8.4)
**Hypertension/ cardiovascular disease**									
No	87	Reference 1.0	Reference 1.0	Reference 1.0	Reference 1.0	Reference 1.0	Reference 1.0	Reference 1.0	Reference 1.0
Yes	99	-7.9* (-15.7 to -0.7)	0.5 (-12.4 to 13.4)	-7.4 (-15.8 to 1.0)	0.0 (-6.5 to 6.5)	2.3 (-4.9 to 9.5)	1.2 (-7.1 to 9.5)	0.8 (-11.6 to 13.2)	1.95 (-4.1 to 7.9)
**Chronic lung disease**									
No	144	Reference 1.0	Reference 1.0	Reference 1.0	Reference 1.0	Reference 1.0	Reference 1.0	Reference 1.0	Reference 1.0
Yes	42	-7.0 (-16.3 to 2.4)	-15.1 (-30.2 to -0.0)	-5.4 (-15.5 to 4.6)	-7.2 (-14.8 to 0.4)	-9.7* (-18.1 to -1.3)	-6.0 (-15.8 to 3.9)	-20.5* (-34.8 to -6.1)	-2.6 (-9.7 to 4.4)
**Ventilation support**									
HFNO/NIV	95	Reference 1.0	Reference 1.0	Reference 1.0	Reference 1.0	Reference 1.0	Reference 1.0	Reference 1.0	Reference 1.0
invasive ventilation	91	-5.6 (-13.4 to 2.3)	-12.4 (-25.1 to 0.4)	-12.3* (-20.6 to -4.0)	-2.2 (-8.6 to 4.3)	-1.4 (-8.6 to 5.8)	-7.8 (-16.0 to 0.5)	-1.26 (-13.7 to 11.2)	2.8 (-3.2 to 8.8)
**In-ICU corticosteroids**									
No	43	Reference 1.0	Reference 1.0	Reference 1.0	Reference 1.0	Reference 1.0	Reference 1.0	Reference 1.0	Reference 1.0
Yes	143	-3.6 (-12.9 to 5.7)	5.7 (-9.4 to 20.8)	3.3 (-6.6 to 13.3)	1.9 (-5.7 to 9.5)	3.6 (-4.8 to 12.0)	2.6 (-7.2 to 12.4)	9.1 (-5.4 to 23.6)	6.4 (-0.6 to 13.3)

For peer review only

Abbreviations: HFNO=high flow nasal oxygen; NIV=non-invasive ventilation. *Statistically significant p-value <0.05.

For peer review only

STROBE (Strengthening The Reporting of OBservational Studies in Epidemiology) Checklist

A checklist of items that should be included in reports of observational studies. You must report the page number in your manuscript where you consider each of the items listed in this checklist. If you have not included this information, either revise your manuscript accordingly before submitting or note N/A.

Note: An Explanation and Elaboration article discusses each checklist item and gives methodological background and published examples of transparent reporting. The STROBE checklist is best used in conjunction with this article (freely available on the Web sites of PLoS Medicine at http://www.plosmedicine.org/, Annals of Internal Medicine at http://www.annals.org/, and Epidemiology at http://www.epidem.com/). Information on the STROBE Initiative is available at www.strobe-statement.org.

Section and Item	Item No.	Recommendation	Reported on Page No.
**Title and Abstract**	1	(*a*) Indicate the study's design with a commonly used term in the title or the abstract	
		(*b*) Provide in the abstract an informative and balanced summary of what was done and what was found	
**Introduction**			
Background/Rationale	2	Explain the scientific background and rationale for the investigation being reported	
Objectives	3	State specific objectives, including any prespecified hypotheses	
**Methods**			
Study Design	4	Present key elements of study design early in the paper	
Setting	5	Describe the setting, locations, and relevant dates, including periods of recruitment, exposure, follow-up, and data collection	
Participants	6	(*a*) *Cohort study*—Give the eligibility criteria, and the sources and methods of selection of participants. Describe methods of follow-up<br>*Case-control study*—Give the eligibility criteria, and the sources and methods of case ascertainment and control selection. Give the rationale for the choice of cases and controls<br>*Cross-sectional study*—Give the eligibility criteria, and the sources and methods of selection of participants	
		(*b*) *Cohort study*—For matched studies, give matching criteria and number of exposed and unexposed<br>*Case-control study*—For matched studies, give matching criteria and the number of controls per case	
Variables	7	Clearly define all outcomes, exposures, predictors, potential confounders, and effect modifiers. Give diagnostic criteria, if applicable	

For peer review only

Section and Item	Item No.	Recommendation	Reported on Page No.
Data Sources/ Measurement	8*	For each variable of interest, give sources of data and details of methods of assessment (measurement). Describe comparability of assessment methods if there is more than one group	
Bias	9	Describe any efforts to address potential sources of bias	
Study Size	10	Explain how the study size was arrived at	
Quantitative Variables	11	Explain how quantitative variables were handled in the analyses. If applicable, describe which groupings were chosen and why	
Statistical Methods	12	(*a*) Describe all statistical methods, including those used to control for confounding	
		(*b*) Describe any methods used to examine subgroups and interactions	
		(*c*) Explain how missing data were addressed	
		(*d*) *Cohort study*—If applicable, explain how loss to follow-up was addressed<br>*Case-control study*—If applicable, explain how matching of cases and controls was addressed<br>*Cross-sectional study*—If applicable, describe analytical methods taking account of sampling strategy	
		(*e*) Describe any sensitivity analyses	
**Results**			
Participants	13*	(a) Report numbers of individuals at each stage of study—eg numbers potentially eligible, examined for eligibility, confirmed eligible, included in the study, completing follow-up, and analysed	
		(b) Give reasons for non-participation at each stage	
		(c) Consider use of a flow diagram	
Descriptive Data	14*	(a) Give characteristics of study participants (eg demographic, clinical, social) and information on exposures and potential confounders	
		(b) Indicate number of participants with missing data for each variable of interest	
		(c) *Cohort study*—Summarise follow-up time (eg, average and total amount)	
Outcome Data	15*	*Cohort study*—Report numbers of outcome events or summary measures over time	
		Case-control study—Report numbers in each exposure category, or summary measures of exposure	
		Cross-sectional study—Report numbers of outcome events or summary measures	

For peer review only

Section and Item	Item No.	Recommendation	Reported on Page No.
Main Results	16	(*a*) Give unadjusted estimates and, if applicable, confounder-adjusted estimates and their precision (eg, 95% confidence interval). Make clear which confounders were adjusted for and why they were included	
		(*b*) Report category boundaries when continuous variables were categorized	
		(*c*) If relevant, consider translating estimates of relative risk into absolute risk for a meaningful time period	
Other Analyses	17	Report other analyses done—eg analyses of subgroups and interactions, and sensitivity analyses	
**Discussion**			
Key Results	18	Summarise key results with reference to study objectives	
Limitations	19	Discuss limitations of the study, taking into account sources of potential bias or imprecision. Discuss both direction and magnitude of any potential bias	
Interpretation	20	Give a cautious overall interpretation of results considering objectives, limitations, multiplicity of analyses, results from similar studies, and other relevant evidence	
Generalisability	21	Discuss the generalisability (external validity) of the study results	
**Other Information**			
Funding	22	Give the source of funding and the role of the funders for the present study and, if applicable, for the original study on which the present article is based	

*Give information separately for cases and controls in case-control studies and, if applicable, for exposed and unexposed groups in cohort and cross-sectional studies.

Once you have completed this checklist, please save a copy and upload it as part of your submission. DO NOT include this checklist as part of the main manuscript document. It must be uploaded as a separate file.

For peer review only

BMJ Open

Comparing severe COVID-19 outcomes of first and second/third waves: a prospective single-center cohort study of health-related quality of life and pulmonary outcomes 6 months after infection

Journal:	*BMJ Open*
Manuscript ID	bmjopen-2022-071394.R1
Article Type:	Original research
Date Submitted by the Author:	06-Mar-2023
Complete List of Authors:	Darlington, Pernilla; Karolinska Institute Department of Clinical Science and Education Södersjukhuset, Department of Internal Medicine; Karolinska Institutet, Department of Clinical Science and Education<br>Roël , Mari; Karolinska Institute Department of Clinical Science and Education Södersjukhuset, Department of Internal Medicine<br>Cronhjort , Maria; Karolinska Institutet, Department of Clinical Science and Education<br>Hanna, Gabriel; Karolinska Institute Department of Clinical Science and Education Södersjukhuset<br>Hedman , Anders; Karolinska Institutet, Department of Clinical Science and Education<br>Joelsson-Alm , Eva; Karolinska Institutet, Department of Clinical Science and Education<br>Schandl, Anna; Karolinska Institutet, Department of Clinical Science and Education
**Primary Subject Heading**:	Anaesthesia
Secondary Subject Heading:	Respiratory medicine, Infectious diseases
Keywords:	COVID-19, Quality of Life, Chest imaging < RADIOLOGY & IMAGING

SCHOLARONE™
Manuscripts

I, the Submitting Author has the right to grant and does grant on behalf of all authors of the Work (as defined in the below author licence), an exclusive licence and/or a non-exclusive licence for contributions from authors who are: i) UK Crown employees; ii) where BMJ has agreed a CC-BY licence shall apply, and/or iii) in accordance with the terms applicable for US Federal Government officers or employees acting as part of their official duties; on a worldwide, perpetual, irrevocable, royalty-free basis to BMJ Publishing Group Ltd ("BMJ") its licensees and where the relevant Journal is co-owned by BMJ to the co-owners of the Journal, to publish the Work in this journal and any other BMJ products and to exploit all rights, as set out in our licence.

The Submitting Author accepts and understands that any supply made under these terms is made by BMJ to the Submitting Author unless you are acting as an employee on behalf of your employer or a postgraduate student of an affiliated institution which is paying any applicable article publishing charge ("APC") for Open Access articles. Where the Submitting Author wishes to make the Work available on an Open Access basis (and intends to pay the relevant APC), the terms of reuse of such Open Access shall be governed by a Creative Commons licence – details of these licences and which Creative Commons licence will apply to this Work are set out in our licence referred to above.

Other than as permitted in any relevant BMJ Author's Self Archiving Policies, I confirm this Work has not been accepted for publication elsewhere, is not being considered for publication elsewhere and does not duplicate material already published. I confirm all authors consent to publication of this Work and authorise the granting of this licence.

For peer review only

2022-12-26

Comparing severe COVID-19 outcomes of first and second/third waves: a prospective single-center cohort study of health-related quality of life and pulmonary outcomes 6 months after infection

Authors: Pernilla DARLINGTON[1,2]*, Mari ROËL[1]*, Maria CRONHJORT[2,3], Gabriel HANNA[4], Anders HEDMAN[2,5], Eva JOELSSON-ALM[2,3], Anna SCHANDL[2,3].

*Shared first authorship

Affiliations: [1]Department of Internal Medicine, Södersjukhuset, Stockholm; [2]Department of Clinical Science and Education, Södersjukhuset, Karolinska Institutet, Sweden; [3]Department of Anesthesia and intensive care, Södersjukhuset Stockholm, Sweden; [4]Department of Radiology, Södersjukhuset, Stockholm, Sweden; [5]Department of Cardiology, Södersjukhuset Stockholm, Sweden.

Corresponding author: Pernilla Darlington, Department of Internal Medicine, Södersjukhuset, Stockholm, Sjukhusbacken 10, 118 83. Stockholm, e-mail address: pernilla.darlington@ki.se

Keywords: COVID-19, CT, quality of life, chest imaging

For peer review only

2022-12-26

ABSTRACT

Objective

To clarify if outcomes have improved with time in intensive care unit (ICU) survivors treated for severe COVID-19, comparing first with second/third waves.

Design

Prospective cohort study.

Setting

Single-center study of adult COVID-19 patients with respiratory distress admitted to two Swedish ICUs during the first wave (1 March– 1 September 2020) and second/third waves (2 September 2020– 1 August 2021).

Participants

456 critically ill COVID-19 patients who survived for at least 90 days.

Main outcome measures

Outcomes were differences in health-related quality of life (HRQL) and pulmonary function between waves. General linear regression and multivariable logistic regression were used to present mean score differences (MSD) and odds ratios (OR) with 95% confidence intervals (CI).

Results

Among the 456 (67%) critically ill COVID-19 patients who survived for at least 90 days after ICU discharge, 278 (61%) attended the follow-up approximately 6 months after ICU discharge. HRQL was similar between the groups, except that the second/third wave

For peer review only - http://bmjopen.bmj.com/site/about/guidelines.xhtml

2022-12-26

survivors scored higher in the role physical domain (MSD 20.2, 95%CI: 7.3-33.1) and lower on bodily pain (MSD 12.2, 95%CI: 3.6-20.8). On the other hand, first wave survivors had better diffusion capacity for carbon monoxide (DLCO) (OR 1.9, 95%CI: 1.1-3.5).

Conclusions

This study indicates that even though intensive care treatment strategies have changed with time, long-term HRQL and impaired respiratory health remain similar at 6 months for patients surviving severe COVID-19 in the first and second/third waves of the pandemic.

Strengths and limitations of this study

*The main methodological strengths of the study lie in the prospective and comprehensive data collection of long-term outcomes in critically ill patients with COVID-19 together with the use of well-validated outcome tests and questionnaires.

*Limitations are the risk of selection bias as some COVID-19 survivors opted out of the follow-up program. Though, when comparing baseline data for all survivors, no statistically significant differences were found. Further survival rate and follow-up rate were higher for those from the first wave.

*Since data on virus strains were not available, we could not adjust for this parameter and potential confounding effects cannot be ruled out.

For peer review only - http://bmjopen.bmj.com/site/about/guidelines.xhtml

2022-12-26

INTRODUCTION

Coronavirus disease 2019 (COVID-19), caused by severe acute respiratory syndrome coronavirus 2 (SARS-CoV-2), was declared a global pandemic in March 2020 and has resulted in numerous severely ill individuals in need of respiratory support[1] and millions of deaths worldwide[2]. The majority survived the acute episode of a critical illness but were confronted with physical and psychological sequelae after discharge from hospital which prolonged recovery[3]. Approximately 35-80% of the survivors experienced persistent symptoms of fatigue, dyspnea, joint pain, sleep disorders, and loss of memory and concentration lasting beyond 4 weeks after a moderate to severe acute infection, which is referred to as "post-acute COVID-19 syndrome"[4-6] Long-lasting respiratory complications as well as other functional deficits which may cause substantial morbidity have been reported among intensive care unit (ICU) survivors in the first year after hospital discharge[7-10]. Furthermore, a high number of residual abnormalities on chest CT scan have been found several months after discharge from hospital, and acute respiratory distress syndrome has been associated with more severe radiological findings[11]. Since the start of the pandemic the risk for hospital admission has varied as new variants of SARS-CoV-2 have emerged [12]. Likewise, worldwide different virus variants have been dominating the waves in Sweden [13]. Further, during the second and third waves, scientific and clinical understanding of the illness improved, and potentially more effective treatments were provided. Yet, whether functional outcomes have improved in line with the increase in knowledge about COVID-19 remains unexplored. Therefore, this prospective single-center cohort study aimed to clarify whether in ICU survivors treated for severe COVID-19 infection comparing first with second/third waves if outcomes have improved with time. Further, to identify factors associated with worse long-term outcomes.

For peer review only - http://bmjopen.bmj.com/site/about/guidelines.xhtml

2022-12-26

METHODS

Study design and setting

This was a prospective, single-centre cohort study including all patients with acute COVID-19 infection who were admitted to two ICUs at Södersjukhuset, Stockholm, Sweden between 1 March 2020 and 1 August 2021. In Sweden, the first cases of severely ill patients were admitted to the ICU at the beginning of March 2020 with a peak in April and then subsided during the summer [14]. Therefore, this time period has been considered to separate the end of the first wave [15]. The beginning of the second wave was estimated to start on 2 September 2020. Thereafter under a longer period, there was an increase in cases with one peak around new year and one peak in April 2021. There was no clear gap in time between the second and third waves, even if there was a clear decrease in cases in March 2021 [14], and the second and third waves were therefore grouped as one time period in this study. Thus, the study was categorized into two time periods: the first wave (1 March–1 September 2020) and the second/third waves (2 September 2020–1 August 2021). The main differences in treatment strategies between the two time periods were that corticosteroids became standard treatment in the ICU at the end of the first wave. Also, there was a shift from early invasive ventilation to allowing non-invasive ventilation during the second/third waves.

Before the pandemic, the two ICUs had a total number of 16 beds. During the first wave, the number of ICU beds expanded to a maximum of 60 beds in April and May 2020. At the beginning of the second wave, the number of ICU beds were 21, rising to 29 beds in November 2020 and further up to maximum of 33 beds in April and May 2021. At the end of the third wave, the number of beds were 21. The nurse:patient ratio was 1:1-2 and the physician:patient ratio 1:4-6 during all three waves, but the proportion of intensive care

For peer review only - http://bmjopen.bmj.com/site/about/guidelines.xhtml

2022-12-26

specialized nurses and physicians decreased during the peaks of the pandemic. None of the ICUs were exclusive for Covid-19 patients.

The admission criteria to the ICU due to COVID-19 were need of respiratory support (ie HFNO/NIV/invasive ventilation). During second/third wave there was a step down for stable patients on high-flow treatment with oxygen (HFNO), but still under continues observation. This whole period was included in the length of ICU stay. Since the vaccination against COVID-19 in Stockholm started with the oldest and most fragile first and for adults ≥75 at the end of March close to the peak of the third wave [16], this is believed to have had little impact on the cohort. The second wave was dominated by a non-variant of concern (VOC) of SARS-CoV-2 virus associated with less severe disease in a Swedish study in comparison to the Alpha VOC that dominated the third wave [13].

Ethical considerations

Study approval was granted from the Swedish Ethical Review Authority (Diarie number: 2020-03760) and written informed consent was obtained from all included participants.

Participants

All critically ill patients with a positive polymerase chain reaction test for COVID-19 and who were treated for respiratory failure with invasive ventilation, HFNO or non-invasive ventilation (NIV) in the ICU were eligible for inclusion in the study. Surviving patients were invited for a follow-up in the hospital at six months after hospital discharge. Together with the invitation for follow-up, a questionnaire assessing health-related quality of life was sent

For peer review only - http://bmjopen.bmj.com/site/about/guidelines.xhtml

2022-12-26

to the patients. At follow-up the patients met a nurse and a physician for recapitulation of their ICU stay. The patients were invited on separate occasions for pulmonary function testing and a chest CT scan. Exclusion criteria as shown in Figure 1 were deceased within 90 days after ICU admission, follow-up in another hospital being referred to or judged not to be able to participate (i.e. no home address or impaired cognition).

Data Collection

Data on demographic and clinical details were recorded from medical charts regarding age, sex, body mass index (BMI), smoking habits, comorbidities, Simplified Acute Physiological Score III (SAPS 3; a scoring system used to predict mortality risk in the ICU, where a higher score indicates a higher mortality risk [17]), corticosteroid treatment, respiratory support (invasive/NIV/HFNO) and ICU length of stay.

Outcomes

The outcomes were health-related quality of life (HRQL) and pulmonary impairments on chest CT scan, and capacity for gas-exchange as a measurement of pulmonary function approximately 6 months after ICU discharge between the first and second/third waves.

The Medical Outcome Study (MOS) Short Form-36 (SF-36) [18] is the most commonly used generic instrument for measuring health-related quality of life (HRQL). In this study HRQL was assessed with a version of this instrument, the 36-item questionnaire RAND-36 Item Health Survey (RAND Corp). SF-36 and RAND-36 are similar, except for some minor differences, e.g. RAND-36 lacks an authorized algorithm for calculation of Mental and

For peer review only - http://bmjopen.bmj.com/site/about/guidelines.xhtml

Physical Component Summary scores [19].The Swedish translation (RAND-36 version 1.02) has been shown to be equivalent to the English[19]. It includes reference data for norm-based comparisons and has been shown to be an acceptable and reliable instrument of measuring HRQL in a Swedish general population [20]. The Swedish version of RAND-36 questionnaire includes the following eight domains: physical functioning (10 items), role physical (4 items), bodily pain (2 items), general health (5 items), vitality (4 items), social functioning (2 items), role emotional (3 items) and emotional well-being (5 items)[21]. Questionnaire responses were linearly transformed into scores between 0 and 100, where a higher score represents a higher HRQL[19, 21]. Minimally clinically important differences were set to 3-5 point differences between groups[22]. Missing items were accounted for with automatic calculation of the scores. If more than half of the scores in one domain were missing, no result was presented.

Pulmonary impairments were identified with a CT scan of the thoracic region using a Siemens Somatom Drive with a standard 120-kV CT thorax protocol as described in a previous publication[7] . International standard thoracic radiological terminology from the Fleischner Society[23] was used to identify the parenchymal changes related to SARS-CoV-2 infection,[24] such as ground-glass opacities, subpleural bands, reticular pattern, and bronchiectasis. A semi-quantitative CT score (CT involvement score) was estimated based on lobar involvement and then calculated as a total score for all five lobes: 0: 0%, 1: <5%, 2: 5%–25%, 3: 26%–50%, 4: 51%–75%, 5: >75%, yielding a score ranging from 0 to 5 in each lobe and a global score of 0–25[25].

The capacity for gas exchange, i.e., diffusion capacity for carbon monoxide (DLCO) (uncorrected value), was measured according to standard methods[26]. DLCO values were

For peer review only - http://bmjopen.bmj.com/site/about/guidelines.xhtml

2022-12-26

expressed in accordance with reference values (Hedenström)[27, 28] with DLCO <80% as cut-off for impaired pulmonary function[8, 9, 11, 29].

Further, to investigate potential factor's role for the long-term outcome for HRQL and pulmonary impairment, following were included: age, sex, comorbidities (i.e. diabetes, hypertension/cardiovascular disease, chronic lung disease), ventilation support and in ICU-corticosteroids. All known to influence the course of the disease in COVID-19 [30-32].

Statistical analyses

Multivariable general linear regression models were used to estimate mean score differences (MSD) with 95% CI between groups and when analyzing the role of patients or clinical characteristics for RAND-36 scores. Multivariable logistic regression models were used to assess lung function changes between first and second/third waves and the role of patients or clinical characteristics, presented as odds ratios (OR) with 95% confidence intervals (CI). For the multivariate analysis comparing waves, confounders other than age (≤50, 50-65, >65 years) and sex (male/female), in this case tobacco smoking habits (ever/never) and chronic lung disease (yes/no) were chosen since they are known as risk factors for a reduced lung function [33]. The other models were adjusted for age (≤65/>65 years), sex (male/female), comorbidity (diabetes, hypertension/cardiovascular disease, and chronic lung disease), corticosteroids (yes/no) and invasive ventilation (yes/no). Two separate statistical analyses were conducted including patients with at least one test result, and patients who had complete data on the three examinations (chest CT scan, pulmonary function test and questionnaires). Since the study was part of a follow-up program after ICU treatment with extra investigations for those with respiratory distress due to COVID-19

For peer review only - http://bmjopen.bmj.com/site/about/guidelines.xhtml

2022-12-26

during the first wave and later extended to also include second/third wave survivors, the sample size was not calculated in advance. Statistical analyses were performed with Graph Pad Prism 8 (Jamovi version 2.3.19) and an experienced biostatistician validated all analyses.

Patient and public involvement

Since the hospital was under heavy burden due to COVID-19 and the follow-up had to be organized within a short time, not knowing what to expect, patients and members of public health were not involved in the set-up of the study.

For peer review only

For peer review only - http://bmjopen.bmj.com/site/about/guidelines.xhtml

2022-12-26

RESULTS

Participants

In total, 678 patients were treated for severe COVID-19 infection during the study period, among which 456 (67%) survived for at least 90 days after ICU discharge. Of these, 278 (61%) attended the follow-up evaluation approximately 6 months after ICU discharge (Figure 1). First-wave survivors were younger, had lower BMI, fewer cardiovascular diseases, higher CRP max, and were more often treated with invasive ventilation, but less frequently with corticosteroids (Table 1). Among survivors with chronic lung disease, the vast majority had asthma. Patient characteristics and ICU-related data were similar between survivors who attended the follow-up and non-participants (Supplementary Table 1), but among non-responders who declined follow-up, non-invasive ventilation was more common (Supplementary Table 2). Further, distribution of age, sex, comorbidities, corticosteroid treatment and type of ventilation were similar between participants who completed the questionnaires and performed the pulmonary function test and chest CT scan and those who did not (Supplementary Table 3). Median time to follow-up with chest CT scan was 6 months (IQR 5-7) and for pulmonary function test 6 months (IQR 6-7), with no significant differences between waves.

For peer review only - http://bmjopen.bmj.com/site/about/guidelines.xhtml

2022-12-26

Table 1. Characteristics of included COVID-19 intensive care unit survivors across the first and second/third pandemic waves.

	**First wave** (1 March–1 Sept 2020)	**Second/third waves** (2 Sept 2020 – 1 Aug 2021)	**p-value**
Total number, n	122	156	
Follow-up time, months, Md (IQR)	5 (4-5)	6 (5-8)	<0.001*
Age, years Md (IQR)	58 (50-66)	61 (53-69)	<0.05*
Male n (%)	93 (76)	110 (71)	0.34
BMI, kg/m^2 Md (IQR)	28 (25-31)	29 (26-33)	<0.05*
Diabetes n (%)	24 (20)	36 (23)	0.56
Hypertension n (%)	53 (43)	72 (46)	0.72
Cardiovascular disease n (%)	12 (10)	30 (19)	<0.05*
Chronic lung disease n (%)	22 (18)	37 (24)	0.30
Ever smoker n (%)	46 (38)	74 (47)	0.11
CRP max, mg/L, Md (IQR)	310 (199-355)	182 (126-248)	<0.001*
SAPS 3, Md (IQR)	54 (50-60)	56 (52-59)	0.52
Invasive ventilation n (%)	72 (59)	64 (41)	<0.01*
Corticosteroid treatment n (%)	49 (40)	156 (100)	<0.001*
Length of ICU stay (days), Md (IQR)	13 (4-23)	9 (6-16)	0.33

BMI= body mass index; CRP = C reactive protein; IQR=Interquartile range; Md=Median; SAPS= Simplified Acute Physiology Score (missing information in 37 participants); *p value <0.05.

HRQL

Among the 187 ICU survivors who reported HRQL data, 76 were treated in the ICU for COVID-19 during the first wave. First-wave survivors reported lower scores in all HRQL domains, with clinically relevant and statistically significant differences in the role physical domain (MSD 20.2, 95%CI: 7.3 to 33.1), bodily pain (MSD 12.2, 95%CI: 3.6 to 20.8) and general health (MSD 7.2, 95%CI: 0.7 to 13.6) (Table 2). Similar results were found for

For peer review only - http://bmjopen.bmj.com/site/about/guidelines.xhtml

2022-12-26

survivors of COVID-19 with complete case data, except for non-significant results for general health (MSD 6.9, 95%CI: -1.3 to 15.2).

Table 2. Health-related quality of life (HRQL) comparing COVID-19 intensive care unit survivors across the first and second/third waves, presented as mean score differences (MSD) with 95% confidence intervals (CI).

Health-related quality of life domains	**First wave**	**Second/third waves**	**Unadjusted analysis** Mean score differences (95%CI)	**Adjusted analysis** Mean score differences (95%CI)
	Mean scores (95%CI)	Mean scores (95%CI)		
Total number	76	111		
Physical function	62 (57-68)	64 (58-69)	1.3 (-6.7 to 9.3)	3.6 (-4.2 to 11.5)
Role physical	31 (22-40)	50 (41-58)	18.5* (5.6 to 31.3)	20.2* (7.3-33.1)
Bodily pain	59 (52-66)	69 (64-75)	10.8* (2.3 to 19.3)	12.2* (3.6 to 20.8)
General health	49 (44-54)	56 (51-60)	6.3 (-0.2 to 12.8)	7.2* (0.7 to 13.6)
Vitality	47 (41-52)	53 (48-58)	6.3 (-0.9 to 13.5)	7.0 (-0.2 to 14.2)
Social function	62 (56-68)	67 (62-73)	5.6 (-2.9 to 14.0)	6.3 (-2.2 to 14.7)
Role emotional	58 (48-68)	68 (60-75)	9.9 (-2.6 to 22.5)	12.0 (-0.5 to 24.6)
Mental health	67 (62-72)	74 (70-78)	6.8* (0.8 to 12.8)	6.9 (-0.8 to 13.1)

The model was adjusted for age, sex, tobacco smoking, chronic lung disease. *Statistically significant p-value <0.05.

In the multivariate analysis, older age (MSD -12.9, 95%CI: -21.0 to -4.8) was associated with worse physical function and similar was seen for those with diabetes (MSD -11.0, 95%CI: -21.0 to -1.1). Diabetes also associated with lower scores on general health (MSD -11.6, 95%CI: -20.0 to -3.3) and mental health (MSD -9.5, 95%CI: -17.3 to -1.7). Survivors treated with invasive ventilation were more likely to report lower scores on bodily pain (MSD -11.8, 95%CI: -20.2 to -3.4). Lower scores on role emotional were seen for those with chronic lung

For peer review only - http://bmjopen.bmj.com/site/about/guidelines.xhtml

2022-12-26

disease (MSD -24.2, 95%CI: -39.7 to -8.8) (Table 3). The univariate analyses can be found in Supplementary Table 4. Similar results were seen in the complete case analysis, where also diabetes associated with more bodily pain (MSD -16.4, 95%CI: -29.5 to -3.3) but not with lower scores for mental health (MSD -8.9, 95%CI: -18.8 to 1.0).

Table 3. Multivariate analysis of patient- and clinical characteristics in relation to health- related quality of life (HRQL) in COVID-19 intensive care unit survivors presented as adjusted mean score differences (MSD) with 95% confidence intervals (CI).

**Variables**	**Physical function** MSD (95%CI)	**Role physical** MSD (95%CI)	**Bodily pain** MSD (95%CI)	**General health** MSD (95%CI)	**Vitality** MSD (95%CI)	**Social function** MSD (95%CI)	**Role emotional** MSD (95%CI)	**Mental health** MSD (95%CI)
**Age, years**<br>≤65<br>>65	Reference 1.0<br>-12.9*<br>(-21.0 to -4.8)	Reference 1.0<br>-1.5<br>(-15.0 to 12.0)	Reference 1.0<br>-5.3<br>(-14.0 to 3.5)	Reference 1.0<br>-2.2<br>(-9.0 to 4.7)	Reference 1.0<br>2.4<br>(-5.3 to 10.0)	Reference 1.0<br>-2.6<br>(-11.6 to 6.3)	Reference 1.4<br>1.7<br>(-11.8 to 14.7)	Reference 1.0<br>1.5<br>(-4.8 to 7.9)
**Sex**<br>Female<br>Male	Reference 1.0<br>5.7<br>(-3.2 to 14.6)	Reference 1.0<br>11.1<br>(-3.6 to 25.8)	Reference 1.0<br>4.7<br>(-4.8 to 14.2)	Reference 1.0<br>4.2<br>(-3.2 to 11.6)	Reference 1.0<br>6.6<br>(-1.7 to 15.0)	Reference 1.0<br>9.8<br>(-0.1 to 19.5)	Reference 1.0<br>-4.2<br>(-18.6 to 10.2)	Reference 1.0<br>1.4<br>(-5.5 to 8.4)
**Diabetes**<br>No<br>Yes	Reference 1.0<br>-11.0*<br>(-21.0 to -1.1)	Reference 1.0<br>-8.5<br>(-25.1 to 8.1)	Reference 1.0<br>-9.2<br>(-19.9 to 1.5)	Reference 1.0<br>-11.6*<br>(-20.0 to -3.3)	Reference 1.0<br>-6.3<br>(-13.2 to 9.9)	Reference 1.0<br>-5.6<br>(-16.5 to 5.3)	Reference 1.0<br>-11.5<br>(-27.6 to 4.6)	Reference 1.0<br>-9.5*<br>(-17.3 to -1.7)
**Hypertension/ cardiovascular disease**<br>No<br>Yes	Reference 1.0<br>-3.2<br>(-11.5 to 5.3)	Reference 1.0<br>-1.1<br>(-15.1 to 13.0)	Reference 1.0<br>-6.4<br>(-15.5 to 2.6)	Reference 1.0<br>2.4<br>(-4.7 to 9.4)	Reference 1.0<br>1.7<br>(-6.2 to 9.6)	Reference 1.0<br>1.1<br>(-8.1 to 10.4)	Reference 1.0<br>1.2<br>(-12.6 to 14.9)	Reference 1.0<br>3.4<br>(-3.3 to 1.0)
**Chronic lung disease**<br>No<br>Yes	Reference 1.0<br>-5.8<br>(-15.5 to 3.8)	Reference 1.0<br>-13.6<br>(-29.4 to 2.2)	Reference 1.0<br>-5.6<br>(-15.9 to 4.6)	Reference 1.0<br>-6.9<br>(-14.9 to 1.1)	Reference 1.0<br>-8.3<br>(-17.3 to 0.7)	Reference 1.0<br>-2.9<br>(-13.4 to 7.4)	Reference 1.0<br>-24.2*<br>(-39.7 to -8.8)	Reference 1.0<br>-3.6<br>(-11.0 to 3.9)
**Ventilation support**<br>HFNO/NIV<br>invasive ventilation	Reference 1.0<br>-6.7<br>(-14.6 to 1.1)	Reference 1.0<br>-8.5<br>(-20.6 to 5.4)	Reference 1.0<br>-11.8*<br>(-20.2 to -3.4)	Reference 1.0<br>-1.6<br>(-8.2 to 4.9)	Reference 1.0<br>0.4<br>(-7.7 to 7.0)	Reference 1.0<br>-7.5<br>(-16.1 to 1.0)	Reference 1.0<br>0.5<br>(-12.2 to 13.2)	Reference 1.0<br>3.3<br>(-2.9 to 9.4)
**In-ICU corticosteroids**<br>No<br>Yes	Reference 1.0<br>-6.1<br>(-18.3 to 6.1)	Reference 1.0<br>-11.9<br>(-32.4 to 8.5)	Reference 1.0<br>-4.5<br>(-17.7 to 8.8)	Reference 1.0<br>-4.6<br>(-14.9 to 5.7)	Reference 1.0<br>-1.7<br>(-13.2 to 9.9)	Reference 1.0<br>-0.6<br>(-14.1 to 12.9)	Reference 1.0<br>3.6<br>(-16.4 to 23.5)	Reference 1.0<br>1.3<br>(-8.3 to 10.9)

Abbreviations: HFNO=high flow nasal oxygen; NIV=non-invasive ventilation; All the variables in the univariate analysis were included in the multivariate analysis with adjustment for time during the pandemic. *Statistically significant p-value <0.05.

For peer review only

For peer review only - http://bmjopen.bmj.com/site/about/guidelines.xhtml

2022-12-26

Pulmonary outcomes

Compared with first wave survivors, patients in the second/third waves had worse pulmonary function with lower DLCO (OR 1.9, CI: 1.1-3.5) (Table 4). Similar findings were seen in patients who had complete data from the three examinations.

Table 4. Odds ratios (OR) with 95% confidence intervals (CI) of CT score and diffusion capacity (DLCO) comparing COVID-19 intensive care unit survivors across the first and second/third pandemic waves. First wave patients' data were used as a reference.

Outcomes	**First wave**		**Second/third waves**		**Unadjusted model** OR (95%CI)	**Adjusted model** OR (95%CI)
	Numbers	Median (IQR)	Numbers	Median (IQR)		
CT score						
0	25	0	21	0	Reference 1.0	Reference 1.0
≥1	65	7 (5-10)	101	9 (5-13)	1.8 (1.0-3.6)	1.8 (0.9-3.7)
DLCO % of predicted						
≥80	64	96 (90-109)	49	91 (86-96)	Reference 1.0	Reference 1.0
<80	36	68 (62-75)	57	65 (53-71)	2.1 (1.2-3.6)*	1.9 (1.1-3.5)*

Abbreviations: DLCO=diffusion capacity for carbon monoxide; CT=computed tomography; IQR = interquartile range. The models were adjusted for age, sex, tobacco smoking, chronic lung disease. *p-value <0.05.

Factors associated with worse radiological pulmonary outcome (i.e., CT score≥1) were older age (>65 years) (OR 4.5, CI: 1.7-11.8), male sex (OR 2.8, CI: 1.3-6.3) and ICU corticosteroid treatment (OR 4.8, CI: 1.3-17.0) (Table 5). For the complete case analysis, only older age was associated with worse CT scores (OR 3.3, 95%CI: 1.1 to 10.1).

Worse pulmonary function (DLCO<80%) was associated with older age (OR 1.9, CI: 1.0-3.6) and female sex (with female as reference) (OR 0.4, CI: 0.2-0.9) (Table 5), but in the complete case analysis only female sex was associated with worse capacity for gas-exchange (OR 0.3, 95%CI: 0.1 to 0.8).

For peer review only - http://bmjopen.bmj.com/site/about/guidelines.xhtml

Table 5. Patient- and clinical characteristics in relation to pulmonary outcomes in COVID-19 intensive care unit survivors presented as odds ratio (OR) with 95% confidence intervals (CI).

**Variables**	**CT score≥1**			**DLCO <80%**		
	Numbers	OR (95%CI) Univariate	OR (95%CI) Multivariate	Numbers	OR (95%CI) Univariate	OR (95%CI) Multivariate
**Age, years**						
≤65	141	Reference 1.0	Reference 1.0	133	Reference 1.0	Reference 1.0
>65	71	4.3 (1.8-10.7)*	4.5 (1.7-11.8)*	73	2.2 (1.2-3.9)*	1.9 (1.0-3.6)*
**Sex**						
Female	61	Reference 1.0	Reference 1.0	57	Reference 1.0	Reference 1.0
Male	151	1.8 (0.9-3.6)	2.8 (1.3-6.3)*	149	0.5 (0.3-0.9)*	0.4 (0.2-0.9)*
**Diabetes**						
No	166	Reference 1.0	Reference 1.0	141	Reference 1.0	Reference 1.0
Yes	46	1.2 (0.5-2.7)	0.8 (0.3-2.0)	65	1.7 (0.9-3.5)	1.5 (0.7-3.3)
**Hypertension/ cardiovascular disease**						
No	108	Reference 1.0	Reference 1.0	103	Reference 1.0	Reference 1.0
Yes	104	1.4 (0.7-2.7)	1.2 (0.5-2.5)	103	1.8 (1.0-3.2)*	1.5 (0.8-2.9)
**Chronic lung disease**						
No	163	Reference 1.0	Reference 1.0	162	Reference 1.0	Reference 1.0
Yes	49	1.1 (0.5-2.4)	1.6 (0.7-4.0)	44	1.1 (0.6-2.2)	0.8 (0.4-1.8)
**Ventilation support**						
HFNO/NIV	105	Reference 1.0	Reference 1.0	102	Reference 1.0	Reference 1.0
invasive ventilation	107	1.4 (0.7-2.8)	1.4 (0.7-3.0)	104	1.2 (0.7-2.0)	1.3 (0.7-2.5)
**In-ICU corticosteroids**						
No	57	Reference 1.0	Reference 1.0	58	Reference 1.0	Reference 1.0
Yes	155	3.0 (1.5-6.0)*	4.8 (1.3-17.0)*	148	2.5 (1.3-4.9)*	1.7 (0.7-4.2)

Abbreviations: DLCO=diffusion capacity for carbon monoxide; HFNO=high flow nasal oxygen; NIV=non-invasive ventilation; multivariate analysis included all the variables in the univariate analysis and was adjusted for time during the pandemic. *Statistically significant p-value <0.05.

For peer review only

DISCUSSION

In this prospective cohort study of COVID-19 ICU survivors, HRQL and pulmonary outcomes were relatively similar between the pandemic waves, despite a changing patient case-mix, new virus variants and changes in intensive care treatment strategies, except that first wave survivors had better pulmonary function, measured with the capacity for gas-exchange (DLCO) and second/third wave survivors scored higher in the role physical domain and had less bodily pain.

Most evidence indicates a small reduction in mortality rates between the first and second/third pandemic waves among critically ill patients[34, 35]. However, data about mortality trends during the pandemic vary depending on study time frames, outcomes, case-mix and sample size[2]. Only a few studies have investigated whether long-term morbidity follows the mortality results. One of these studies was a multinational study, in which 1000 patients with COVID-19 and severe hypoxaemia were randomized to either 6 mg or 12 mg dexamethasone, but no differences between groups were found in 3-months mortality or HRQL[36].

A systematic review and meta-analysis showed that those with more severe initial illness had poorer long-term recovery after COVID-19 infection[37]. In our study, risk factors for reduced quality of life in at least one parameter at follow-up in ICU survivors treated because of COVID-19 were older age, diabetes, chronic lung disease and invasive ventilation. Diabetes was identified early in the pandemic as a risk factor for critical disease and mortality [38]. Our findings as previously reported with a reduced quality-of-life in the most severely ill COVID-19 survivors compared with the general population [8] are also consistent with data from other populations [31]. Since COVID-19 treatment strategies have improved with time, we hypothesized that patients treated later in the pandemic would have less post-ICU

For peer review only - http://bmjopen.bmj.com/site/about/guidelines.xhtml

morbidity, which was also found for role physical and general health. However, for pulmonary function the outcome was better for first wave survivors. For pulmonary healing, older age and corticosteroid treatment were associated with more residual changes on chest CT scan. Age as a risk factor for severe disease and worse radiological outcome is well-known and has been described previously[9, 39]. So far, few studies have investigated the long-term effects of corticosteroid treatment for pulmonary healing in critically ill patients with COVID-19. Corticosteroid treatment has been used to reduce the inflammation seen in COVID-19 patients. However, recent evidence has shown that even if there may be short-term mortality benefits for mechanically ventilated patients, there may also be negative long-term mortality consequences[40]. A randomized controlled trial showed that corticosteroid treatment improved forced vital capacity in hospitalized patients with COVID-19, but diffusion capacity (DLCO) was not included in the study[41]. Another study showed that the rate of ventilator-associated pneumonia was higher during the latter part of the pandemic, when the use of corticosteroid treatment had also increased, suggesting different causes of final respiratory failure between the COVID-19 waves[34].

Increasing age and female sex were associated with worse lung function at follow-up. In general, there was a predominance of males who become critically ill[42]. The females in need of ICU treatment in our cohort may have had a worse pulmonary impairment before COVID-19 because there was a discrepancy between CT score and the capacity for gas-exchange (DLCO) in females. An association between female sex and worse DLCO values in hospitalized patients has also been reported by others[29].

For peer review only - http://bmjopen.bmj.com/site/about/guidelines.xhtml

Strength and limitations

The main methodological strengths of the study lie in the prospective and comprehensive data collection together with the use of well-validated outcome tests and questionnaires. The study also has a number of limitations. Firstly, the high number of exclusions due to administrative reasons and as well referral to other hospitals due to lack of beds in the ICU and with own follow-up programs are also to be considered. During this time, the hospital was under heavy burden and all ICU nurses and physicians were needed in bedside work and the follow-up had to be done retrospectively. However, those missed due to administrative reasons were most likely missed at random. Of those who declined follow-up, fewer had needed invasive ventilation and they might have reached complete recovery and considered follow-up as unnecessary. Though, when comparing baseline data for all survivors, no statistically significant differences were found. Further, the results may be biased by the higher survival rate in the first wave which could possibly be explained by change in patient-mix, viral strain, hospital load and differences in treatment strategies, and also the follow-up rate was higher for those in the first wave. In addition, the lack of baseline data prevents identification of new-onset problems. Yet, data on pulmonary function and HRQL is difficult to obtain pre-ICU. The HRQL results were self-reported and may include a risk of response shift. Also, the time interval for follow-up varied which might have had an impact on the results. As HRQL aspects include many variables, and several analyses were conducted, there may be a risk that some statistically significant results may be due to chance. Lastly, results were adjusted for some known confounders. However, since data on virus strains were not available, we could not adjust for this parameter and residual confounding cannot be ruled out.

For peer review only - http://bmjopen.bmj.com/site/about/guidelines.xhtml

For peer review only

This cohort included a heterogenic COVID-19 population who received care at a general hospital in the Stockholm area. We assume that in countries where healthcare organization is similar, the results of this study are likely to be generalizable and may contribute to knowledge on how to assess and accommodate expected problems after severe COVID-19 disease and respiratory distress. This knowledge may also be applicable for other causes of acute respiratory distress syndrome (ARDS) as well as treatment in the ICU of various conditions where, consistent with our findings, functional impairments are common [43, 44]. Given the increasing cumulative number of patients who survive after severe COVID-19 infection, it is important to understand long-term functional outcomes in order to clarify patients' rehabilitation needs and inform development of evidence-based interventions which will help prevent new disabilities. Further research is needed to improve survivorship in ICU patients with COVID-19 infection, and it is of great importance to investigate what factors may negatively affect recovery.

CONCLUSION

This study suggests that even though patient case-mix has changed, new variants of **SARS-**CoV-2 have emerged and intensive care treatment strategies have changed since the start of the pandemic, long-term HRQL and impaired respiratory health remains similar for COVID-19 patients with critical illness.

For peer review only - http://bmjopen.bmj.com/site/about/guidelines.xhtml

DECLARATIONS

Ethics approval and consent to participate

Study approval was granted from the Swedish Ethical Review Authority (Diary number: 2020-03760) and written informed consent was obtained from all included participants.

Declaration of interests

The authors declare that they have no competing interests.

Availability of data and materials

The datasets used and/or analyzed during the current study are available from the corresponding author upon reasonable request.

Funding

Departmental funding only.

Author contributions

PD and MR contributed to conceptualisation, methodology, formal analysis, investigation, writing – original draft. MC contributed to methodology, investigation, writing – review and editing. GH contributed to methodology, investigation, writing – review and editing. AH: conceptualisation, investigation, writing – review and editing. EJA contributed to conceptualisation, formal analysis, writing – review and editing. AS contributed to conceptualisation, methodology, investigation, formal analysis, writing draft – review and editing. All authors read and approved the final manuscript.

For peer review only - http://bmjopen.bmj.com/site/about/guidelines.xhtml

Acknowledgements

We would like to thank all patients who participated in the study and caregivers involved in the follow-up.

For peer review only

For peer review only - http://bmjopen.bmj.com/site/about/guidelines.xhtml

REFERENCES

1. Lim ZJ, Subramaniam A, Ponnapa Reddy M, et al. Case Fatality Rates for Patients with COVID-19 Requiring Invasive Mechanical Ventilation. A Meta-analysis. *Am J Respir Crit Care Med*. 2021; 203: 54-66.
2. Fan G, Yang Z, Lin Q, Zhao S, Yang L and He D. Decreased Case Fatality Rate of COVID-19 in the Second Wave: A study in 53 countries or regions. *Transbound Emerg Dis*. 2021; 68: 213-5.
3. Evans RA, McAuley H, Harrison EM, et al. Physical, cognitive, and mental health impacts of COVID-19 after hospitalisation (PHOSP-COVID): a UK multicentre, prospective cohort study. *Lancet Respir Med*. 2021; 9: 1275-87.
4. Chopra V, Flanders SA, O'Malley M, Malani AN and Prescott HC. Sixty-Day Outcomes Among Patients Hospitalized With COVID-19. *Ann Intern Med*. 2021; 174: 576-8.
5. Carfi A, Bernabei R, Landi F and Gemelli Against C-P-ACSG. Persistent Symptoms in Patients After Acute COVID-19. *JAMA*. 2020; 324: 603-5.
6. Garrigues E, Janvier P, Kherabi Y, et al. Post-discharge persistent symptoms and health-related quality of life after hospitalization for COVID-19. *J Infect*. 2020; 81: e4-e6.
7. Hanna G, Bankler S, Schandl A, et al. The role of ventilatory support for long-term outcomes after critical infection with COVID-19: A prospective cohort study. *Clin Respir J*. 2022; 16: 63-71.
8. Schandl A, Hedman A, Lynga P, et al. Long-term consequences in critically ill COVID-19 patients: A prospective cohort study. *Acta Anaesthesiol Scand*. 2021; 65: 1285-92.
9. Huang C, Huang L, Wang Y, et al. 6-month consequences of COVID-19 in patients discharged from hospital: a cohort study. *Lancet*. 2021; 397: 220-32.
10. Jacobson KB, Rao M, Bonilla H, et al. Patients With Uncomplicated Coronavirus Disease 2019 (COVID-19) Have Long-Term Persistent Symptoms and Functional Impairment Similar to Patients with Severe COVID-19: A Cautionary Tale During a Global Pandemic. *Clin Infect Dis*. 2021; 73: e826-e9.
11. Han X, Fan Y, Alwalid O, et al. Six-month Follow-up Chest CT Findings after Severe COVID-19 Pneumonia. *Radiology*. 2021; 299: E177-E86.
12. Twohig KA, Nyberg T, Zaidi A, et al. Hospital admission and emergency care attendance risk for SARS-CoV-2 delta (B.1.617.2) compared with alpha (B.1.1.7) variants of concern: a cohort study. *Lancet Infect Dis*. 2022; 22: 35-42.
13. Strålin K, Bruce D, Wahlström E, et al. Impact of the Alpha VOC on disease severity in SARS-CoV-2-positive adults in Sweden. *J Infect*. 2022; 84: e3-e5.
14. Ludvigsson JF. How Sweden approached the COVID-19 pandemic: Summary and commentary on the National Commission Inquiry. *Acta Paediatr*. 2023; 112: 19-33.
15. Zhang SX, Arroyo Marioli F, Gao R and Wang S. A Second Wave? What Do People Mean by COVID Waves? - A Working Definition of Epidemic Waves. *Risk Manag Healthc Policy*. 2021; 14: 3775-82.
16. Isitt C, Sjöholm D, Hergens MP, Granath F and Nauclér P. The early impact of vaccination against SARS-CoV-2 in Region Stockholm, Sweden. *Vaccine*. 2022; 40: 2823-7.
17. Metnitz PG, Moreno RP, Almeida E, et al. SAPS 3--From evaluation of the patient to evaluation of the intensive care unit. Part 1: Objectives, methods and cohort description. *Intensive Care Med*. 2005; 31: 1336-44.
18. Ware JE and Sherbourne CD. The MOS 36-item short-form health survey (SF-36). I. Conceptual framework and item selection. *Med Care*. 1992; 30: 473-83.
19. Orwelius L, Nilsson M, Nilsson E, et al. The Swedish RAND-36 Health Survey - reliability and responsiveness assessed in patient populations using Svensson's method for paired ordinal data. *J Patient Rep Outcomes*. 2017; 2: 4.
20. Ohlsson-Nevo E, Hiyoshi A, Noren P, Moller M and Karlsson J. The Swedish RAND-36: psychometric characteristics and reference data from the Mid-Swed Health Survey. *J Patient Rep Outcomes*. 2021; 5: 66.

For peer review only - http://bmjopen.bmj.com/site/about/guidelines.xhtml

21. Hays RD, Sherbourne CD and Mazel RM. The RAND 36-Item Health Survey 1.0. *Health Econ*. 1993; 2: 217-27.
22. Samsa G, Edelman D, Rothman ML, Williams GR, Lipscomb J and Matchar D. Determining clinically important differences in health status measures: a general approach with illustration to the Health Utilities Index Mark II. *Pharmacoeconomics*. 1999; 15: 141-55.
23. Hansell DM, Bankier AA, MacMahon H, McLoud TC, Muller NL and Remy J. Fleischner Society: glossary of terms for thoracic imaging. *Radiology*. 2008; 246: 697-722.
24. Chung M, Bernheim A, Mei X, et al. CT Imaging Features of 2019 Novel Coronavirus (2019-nCoV). *Radiology*. 2020; 295: 202-7.
25. Francone M, Iafrate F, Masci GM, et al. Chest CT score in COVID-19 patients: correlation with disease severity and short-term prognosis. *Eur Radiol*. 2020; 30: 6808-17.
26. Graham BL, Brusasco V, Burgos F, et al. 2017 ERS/ATS standards for single-breath carbon monoxide uptake in the lung. *Eur Respir J*. 2017; 49.
27. Hedenstrom H, Malmberg P and Fridriksson HV. Reference values for lung function tests in men: regression equations with smoking variables. *Ups J Med Sci*. 1986; 91: 299-310.
28. Hedenstrom H, Malmberg P and Agarwal K. Reference values for lung function tests in females. Regression equations with smoking variables. *Bull Eur Physiopathol Respir*. 1985; 21: 551-7.
29. Mendez R, Latorre A, Gonzalez-Jimenez P, et al. Reduced Diffusion Capacity in COVID-19 Survivors. *Ann Am Thorac Soc*. 2021; 18: 1253-5.
30. Dessie ZG and Zewotir T. Mortality-related risk factors of COVID-19: a systematic review and meta-analysis of 42 studies and 423,117 patients. *BMC Infect Dis*. 2021; 21: 855.
31. Rosa RG, Cavalcanti AB, Azevedo LCP, et al. Association between acute disease severity and one-year quality of life among post-hospitalisation COVID-19 patients: Coalition VII prospective cohort study. *Intensive Care Med*. 2023; 49: 166-77.
32. Horby P, Lim WS, Emberson JR, et al. Dexamethasone in Hospitalized Patients with Covid-19. *N Engl J Med*. 2021; 384: 693-704.
33. Raherison C and Girodet PO. Epidemiology of COPD. *Eur Respir Rev*. 2009; 18: 213-21.
34. Carbonell R, Urgeles S, Rodriguez A, et al. Mortality comparison between the first and second/third waves among 3,795 critical COVID-19 patients with pneumonia admitted to the ICU: A multicentre retrospective cohort study. *Lancet Reg Health Eur*. 2021; 11: 100243.
35. Dongelmans DA, Termorshuizen F, Brinkman S, et al. Characteristics and outcome of COVID-19 patients admitted to the ICU: a nationwide cohort study on the comparison between the first and the consecutive upsurges of the second wave of the COVID-19 pandemic in the Netherlands. *Ann Intensive Care*. 2022; 12: 5.
36. Granholm A, Kjaer MN, Munch MW, et al. Long-term outcomes of dexamethasone 12 mg versus 6 mg in patients with COVID-19 and severe hypoxaemia. *Intensive Care Med*. 2022; 48: 580-9.
37. Han Q, Zheng B, Daines L and Sheikh A. Long-Term Sequelae of COVID-19: A Systematic Review and Meta-Analysis of One-Year Follow-Up Studies on Post-COVID Symptoms. *Pathogens*. 2022; 11.
38. Huang I, Lim MA and Pranata R. Diabetes mellitus is associated with increased mortality and severity of disease in COVID-19 pneumonia - A systematic review, meta-analysis, and meta-regression. *Diabetes Metab Syndr*. 2020; 14: 395-403.
39. Chen Y, Ding C, Yu L, et al. One-year follow-up of chest CT findings in patients after SARS-CoV-2 infection. *BMC Med*. 2021; 19: 191.
40. Moreno G, Carbonell R, Martin-Loeches I, et al. Corticosteroid treatment and mortality in mechanically ventilated COVID-19-associated acute respiratory distress syndrome (ARDS) patients: a multicentre cohort study. *Ann Intensive Care*. 2021; 11: 159.
41. Barros C, Freire RS, Frota E, et al. Short-Course of Methylprednisolone Improves Respiratory Functional Parameters After 120 Days in Hospitalized COVID-19 Patients (Metcovid Trial): A Randomized Clinical Trial. *Front Med (Lausanne)*. 2021; 8: 758405.

For peer review only

For peer review only - http://bmjopen.bmj.com/site/about/guidelines.xhtml

42. Peckham H, de Gruijter NM, Raine C, et al. Male sex identified by global COVID-19 meta-analysis as a risk factor for death and ITU admission. *Nat Commun*. 2020; 11: 6317.
43. Herridge MS, Tansey CM, Matté A, et al. Functional disability 5 years after acute respiratory distress syndrome. *N Engl J Med*. 2011; 364: 1293-304.
44. Hofhuis JG, van Stel HF, Schrijvers AJ, Rommes JH and Spronk PE. ICU survivors show no decline in health-related quality of life after 5 years. *Intensive Care Med*. 2015; 41: 495-504.

For peer review only

For peer review only - http://bmjopen.bmj.com/site/about/guidelines.xhtml

Figure legend

Figure 1. Flowchart of study inclusion.

For peer review only

For peer review only - http://bmjopen.bmj.com/site/about/guidelines.xhtml

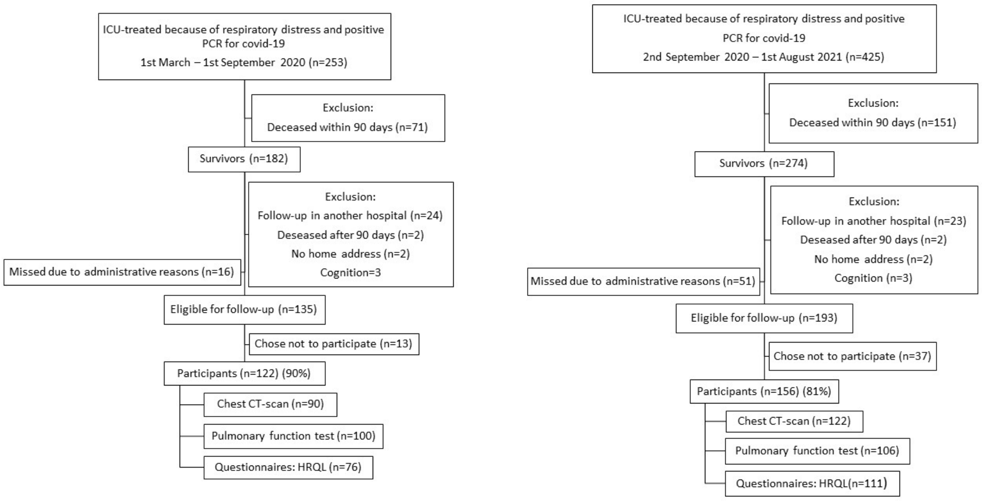

Flowchart of study inclusion.

108x60mm (300 x 300 DPI)

SUPPLEMENTARY FILE

Supplementary Table 1. Comparisons of patient characteristics between participants and non-participants among those invited for follow-up.

Characteristics	Participants	Non-participants	p-values	Missing
Total number, n (%)	278 (85)	50 (15)		
Age, years mean±SD	59.5± 11.9	62.0± 13.7	0.18	
Sex, n (%)<br>Male	203 (73)	37 (74)	0.56	
SAPS 3 score, mean±SD	55.6± 7.8	55.8± 7.6	0.90	51
Ventilator treatment, n (%)<br>Invasive	136 (49)	15 (30)	<0.05	

Student's t-test and Chi$_2$-test were used where appropriate. Statistically significant p-value <0.05.

Supplementary Table 2. Comparisons of patient characteristics between participants and non-participants including all survivors >3 months.

Characteristics	Participants	Non-participants	p-values	Missing
Total number, n (%)	278 (61)	178 (39)		
Age, years mean±SD	59.5± 11.9	61.1± 13.4	0.19	
Sex, n (%)<br>Male	203 (73)	128 (72)	0.83	
SAPS 3 score, mean±SD	55.6± 7.8	55.7± 7.6	0.95	82
Ventilator treatment, n (%)<br>Invasive	136 (49)	89 (50)	0.85	

Student's t-test and Chi$_2$-test were used where appropriate. Statistically significant p-value <0.05.

For peer review only

Supplementary Table 3. Comparison between participants who completed the questionnaires; performed the pulmonary function test; chest CT scan and those who did not.

Measure	Characteristic	Responder	Non-responder	p-value
**RAND-36**		N=187	N=91	
	Age ≥ 65	66 (36)	27 (29)	0.35
	Male	134 (72)	69 (76)	0.46
	Diabetes	39 (21)	21 (23)	0.67
	Hypertension/ cardiovascular disease	99 (53)	40 (44)	0.16
	Chronic lung disease	43 (23)	16 (18)	0.30
	Mechanical ventilation	91 (49)	45 (50)	0.90
	Corticosteroid treatment	143 (76)	62 (68)	0.14
**Pulmonary function test**		N=206	N=72	
	Age ≥ 65	73 (35)	20 (28)	0.24
	Male	149 (72)	54 (75)	0.67
	Diabetes	41 (20)	19 (26)	0.25
	Hypertension/ cardiovascular disease	103 (50)	36 (50)	1.00
	Chronic lung disease	44 (21)	15 (21)	0.92
	Mechanical ventilation	104 (50)	32 (44)	0.38
	Corticosteroid treatment	148 (72)	57 (79)	0.22
**Chest CT scan**		N=212	N=66	
	Age ≥ 65	71 (34)	22 (33)	0.98
	Male	151 (71)	52 (79)	0.23
	Diabetes	46 (22)	14 (21)	0.93
	Hypertension/ cardiovascular disease	104 (49)	35 (53)	0.57
	Chronic lung disease	49 (23)	10 (15)	0.17
	Mechanical ventilation	107 (50)	29 (44)	0.35
	Corticosteroid treatment	155 (73)	50 (76)	0.67

For peer review only

Supplementary Table 4. Univariate analysis of patient- and clinical characteristics in relation to health-related quality of life (HRQL) in COVID-19 intensive care unit survivors presented as adjusted mean score differences (MSD) with 95% confidence intervals (CI).

Variables	Number	**Physical function** MSD (95%CI)	**Role physical** MSD (95%CI)	**Bodily pain** MSD (95%CI)	**General health** MSD (95%CI)	**Vitality** MSD (95%CI)	**Social function** MSD (95%CI)	**Role emotional** MSD (95%CI)	**Mental health** MSD (95%CI)
**Age, years**									
≤65	120	Reference 1.0	Reference 1.0	Reference 1.0	Reference 1.0	Reference 1.0	Reference 1.0	Reference 1.0	Reference 1.0
>65	66	-13.3* (-21.3 to -5.3)	1.0 (-12.5 to 14.4)	-5.2 (-14.1 to 3.6)	-1.1 (-7.9 to 5.6)	3.3 (-4.2 to 10.8)	-1.7 (-10.4 to 7.0)	3.4 (-9.6 to 16.3)	2.5 (-3.7 to 8.8)
**Sex**									
Female	53	Reference 1.0	Reference 1.0	Reference 1.0	Reference 1.0	Reference 1.0	Reference 1.0	Reference 1.0	Reference 1.0
Male	133	6.5 (-2.2 to 15.2)	13.8 (-0.3 to 27.9)	4.6 (-4.7 to 14.0)	5.6 (-1.6 to 12.7)	8.6* (0.7-16.4)	10.2* (1.1 to 19.2)	1.8 (-11.9 to 15.5)	1.8 (-4.8 to 8.4)
**Diabetes**									
No	147	Reference 1.0	Reference 1.0	Reference 1.0	Reference 1.0	Reference 1.0	Reference 1.0	Reference 1.0	Reference 1.0
Yes	39	-11.5* (-21.0 to -2.0)	-6.74 (-22.5 to 9.0)	-10.2 (-20.5 to 0.1)	-10.0* (-17.8 to -2.2)	-4.5 (-13.3 to 4.3)	-3.1 (-13.3 to 7.1)	-10.1 (-25.2 to 5.1)	-8.3* (-15.6 to -1.1)
**Hypertension/ cardiovascular disease**									
No	87	Reference 1.0	Reference 1.0	Reference 1.0	Reference 1.0	Reference 1.0	Reference 1.0	Reference 1.0	Reference 1.0
Yes	99	-7.9* (-15.7 to -0.7)	0.5 (-12.4 to 13.4)	-7.4 (-15.8 to 1.0)	0.0 (-6.5 to 6.5)	2.3 (-4.9 to 9.5)	1.2 (-7.1 to 9.5)	0.8 (-11.6 to 13.2)	1.95 (-4.1 to 7.9)
**Chronic lung disease**									
No	144	Reference 1.0	Reference 1.0	Reference 1.0	Reference 1.0	Reference 1.0	Reference 1.0	Reference 1.0	Reference 1.0
Yes	42	-7.0 (-16.3 to 2.4)	-15.1 (-30.2 to -0.0)	-5.4 (-15.5 to 4.6)	-7.2 (-14.8 to 0.4)	-9.7* (-18.1 to -1.3)	-6.0 (-15.8 to 3.9)	-20.5* (-34.8 to -6.1)	-2.6 (-9.7 to 4.4)
**Ventilation support**									
HFNO/NIV	95	Reference 1.0	Reference 1.0	Reference 1.0	Reference 1.0	Reference 1.0	Reference 1.0	Reference 1.0	Reference 1.0
invasive ventilation	91	-5.6 (-13.4 to 2.3)	-12.4 (-25.1 to 0.4)	-12.3* (-20.6 to -4.0)	-2.2 (-8.6 to 4.3)	-1.4 (-8.6 to 5.8)	-7.8 (-16.0 to 0.5)	-1.26 (-13.7 to 11.2)	2.8 (-3.2 to 8.8)

For peer review only

**In-ICU corticosteroids**									
No	43	Reference 1.0	Reference 1.0	Reference 1.0	Reference 1.0	Reference 1.0	Reference 1.0	Reference 1.0	Reference 1.0
Yes	143	-3.6 (-12.9 to 5.7)	5.7 (-9.4 to 20.8)	3.3 (-6.6 to 13.3)	1.9 (-5.7 to 9.5)	3.6 (-4.8 to 12.0)	2.6 (-7.2 to 12.4)	9.1 (-5.4 to 23.6)	6.4 (-0.6 to 13.3)

Abbreviations: HFNO=high flow nasal oxygen; NIV=non-invasive ventilation. *Statistically significant p-value <0.05.

For peer review only

STROBE (Strengthening The Reporting of OBservational Studies in Epidemiology) Checklist

A checklist of items that should be included in reports of observational studies. You must report the page number in your manuscript where you consider each of the items listed in this checklist. If you have not included this information, either revise your manuscript accordingly before submitting or note N/A.

Note: An Explanation and Elaboration article discusses each checklist item and gives methodological background and published examples of transparent reporting. The STROBE checklist is best used in conjunction with this article (freely available on the Web sites of PLoS Medicine at http://www.plosmedicine.org/, Annals of Internal Medicine at http://www.annals.org/, and Epidemiology at http://www.epidem.com/). Information on the STROBE Initiative is available at www.strobe-statement.org.

Section and Item	Item No.	Recommendation	Reported on Page No.
**Title and Abstract**	1	(*a*) Indicate the study's design with a commonly used term in the title or the abstract	
		(*b*) Provide in the abstract an informative and balanced summary of what was done and what was found	
**Introduction**			
Background/Rationale	2	Explain the scientific background and rationale for the investigation being reported	
Objectives	3	State specific objectives, including any prespecified hypotheses	
**Methods**			
Study Design	4	Present key elements of study design early in the paper	
Setting	5	Describe the setting, locations, and relevant dates, including periods of recruitment, exposure, follow-up, and data collection	
Participants	6	(*a*) *Cohort study*—Give the eligibility criteria, and the sources and methods of selection of participants. Describe methods of follow-up<br>*Case-control study*—Give the eligibility criteria, and the sources and methods of case ascertainment and control selection. Give the rationale for the choice of cases and controls<br>*Cross-sectional study*—Give the eligibility criteria, and the sources and methods of selection of participants	
		(*b*) *Cohort study*—For matched studies, give matching criteria and number of exposed and unexposed<br>*Case-control study*—For matched studies, give matching criteria and the number of controls per case	
Variables	7	Clearly define all outcomes, exposures, predictors, potential confounders, and effect modifiers. Give diagnostic criteria, if applicable	

Section and Item	Item No.	Recommendation	Reported on Page No.
Data Sources/ Measurement	8*	For each variable of interest, give sources of data and details of methods of assessment (measurement). Describe comparability of assessment methods if there is more than one group	
Bias	9	Describe any efforts to address potential sources of bias	
Study Size	10	Explain how the study size was arrived at	
Quantitative Variables	11	Explain how quantitative variables were handled in the analyses. If applicable, describe which groupings were chosen and why	
Statistical Methods	12	(*a*) Describe all statistical methods, including those used to control for confounding	
		(*b*) Describe any methods used to examine subgroups and interactions	
		(*c*) Explain how missing data were addressed	
		(*d*) *Cohort study*—If applicable, explain how loss to follow-up was addressed<br>*Case-control study*—If applicable, explain how matching of cases and controls was addressed<br>*Cross-sectional study*—If applicable, describe analytical methods taking account of sampling strategy	
		(*e*) Describe any sensitivity analyses	
**Results**			
Participants	13*	(a) Report numbers of individuals at each stage of study—eg numbers potentially eligible, examined for eligibility, confirmed eligible, included in the study, completing follow-up, and analysed	
		(b) Give reasons for non-participation at each stage	
		(c) Consider use of a flow diagram	
Descriptive Data	14*	(a) Give characteristics of study participants (eg demographic, clinical, social) and information on exposures and potential confounders	
		(b) Indicate number of participants with missing data for each variable of interest	
		(c) *Cohort study*—Summarise follow-up time (eg, average and total amount)	
Outcome Data	15*	*Cohort study*—Report numbers of outcome events or summary measures over time	
		Case-control study—Report numbers in each exposure category, or summary measures of exposure	
		Cross-sectional study—Report numbers of outcome events or summary measures	

For peer review only

Section and Item	Item No.	Recommendation	Reported on Page No.
Main Results	16	(*a*) Give unadjusted estimates and, if applicable, confounder-adjusted estimates and their precision (eg, 95% confidence interval). Make clear which confounders were adjusted for and why they were included	
		(*b*) Report category boundaries when continuous variables were categorized	
		(*c*) If relevant, consider translating estimates of relative risk into absolute risk for a meaningful time period	
Other Analyses	17	Report other analyses done—eg analyses of subgroups and interactions, and sensitivity analyses	
**Discussion**			
Key Results	18	Summarise key results with reference to study objectives	
Limitations	19	Discuss limitations of the study, taking into account sources of potential bias or imprecision. Discuss both direction and magnitude of any potential bias	
Interpretation	20	Give a cautious overall interpretation of results considering objectives, limitations, multiplicity of analyses, results from similar studies, and other relevant evidence	
Generalisability	21	Discuss the generalisability (external validity) of the study results	
**Other Information**			
Funding	22	Give the source of funding and the role of the funders for the present study and, if applicable, for the original study on which the present article is based	

*Give information separately for cases and controls in case-control studies and, if applicable, for exposed and unexposed groups in cohort and cross-sectional studies.

Once you have completed this checklist, please save a copy and upload it as part of your submission. DO NOT include this checklist as part of the main manuscript document. It must be uploaded as a separate file.

For peer review only

BMJ Open

Comparing severe COVID-19 outcomes of first and second/third waves: a prospective single-center cohort study of health-related quality of life and pulmonary outcomes 6 months after infection

Journal:	*BMJ Open*
Manuscript ID	bmjopen-2022-071394.R2
Article Type:	Original research
Date Submitted by the Author:	18-May-2023
Complete List of Authors:	Darlington, Pernilla; Karolinska Institute Department of Clinical Science and Education Södersjukhuset, Department of Internal Medicine; Karolinska Institutet, Department of Clinical Science and Education<br>Roël , Mari; Karolinska Institute Department of Clinical Science and Education Södersjukhuset, Department of Internal Medicine<br>Cronhjort , Maria; Karolinska Institutet, Department of Clinical Science and Education<br>Hanna, Gabriel; Karolinska Institute Department of Clinical Science and Education Södersjukhuset<br>Hedman , Anders; Karolinska Institutet, Department of Clinical Science and Education<br>Joelsson-Alm , Eva; Karolinska Institutet, Department of Clinical Science and Education<br>Schandl, Anna; Karolinska Institutet, Department of Clinical Science and Education
**Primary Subject Heading**:	Anaesthesia
Secondary Subject Heading:	Respiratory medicine, Infectious diseases
Keywords:	COVID-19, Quality of Life, Chest imaging < RADIOLOGY & IMAGING

SCHOLARONE™
Manuscripts

I, the Submitting Author has the right to grant and does grant on behalf of all authors of the Work (as defined in the below author licence), an exclusive licence and/or a non-exclusive licence for contributions from authors who are: i) UK Crown employees; ii) where BMJ has agreed a CC-BY licence shall apply, and/or iii) in accordance with the terms applicable for US Federal Government officers or employees acting as part of their official duties; on a worldwide, perpetual, irrevocable, royalty-free basis to BMJ Publishing Group Ltd ("BMJ") its licensees and where the relevant Journal is co-owned by BMJ to the co-owners of the Journal, to publish the Work in this journal and any other BMJ products and to exploit all rights, as set out in our licence.

The Submitting Author accepts and understands that any supply made under these terms is made by BMJ to the Submitting Author unless you are acting as an employee on behalf of your employer or a postgraduate student of an affiliated institution which is paying any applicable article publishing charge ("APC") for Open Access articles. Where the Submitting Author wishes to make the Work available on an Open Access basis (and intends to pay the relevant APC), the terms of reuse of such Open Access shall be governed by a Creative Commons licence – details of these licences and which Creative Commons licence will apply to this Work are set out in our licence referred to above.

Other than as permitted in any relevant BMJ Author's Self Archiving Policies, I confirm this Work has not been accepted for publication elsewhere, is not being considered for publication elsewhere and does not duplicate material already published. I confirm all authors consent to publication of this Work and authorise the granting of this licence.

For peer review only

2023-05-09

Comparing severe COVID-19 outcomes of first and second/third waves: a prospective single-center cohort study of health-related quality of life and pulmonary outcomes 6 months after infection

Authors: Pernilla DARLINGTON[1,2]*, Mari ROËL[1,2]*, Maria CRONHJORT[3], Gabriel HANNA[4], Anders HEDMAN[2,5], Eva JOELSSON-ALM[2,6], Anna SCHANDL[2,6].

*Shared first authorship

Affiliations: [1]Department of Internal Medicine, Södersjukhuset, Stockholm; [2]Department of Clinical Science and Education, Södersjukhuset, Karolinska Institutet, Sweden; [3]Department of Clinical Sciences, Danderyd Hospital, Karolinska Institutet, Section of Anesthesiology and Intensive Care, Danderyds sjukhus, SE-182 88, Stockholm, Sweden; [4]Department of Radiology, Södersjukhuset, Stockholm, Sweden; [5]Department of Cardiology, Södersjukhuset Stockholm, Sweden; [6]Department of Anesthesia and intensive care, Södersjukhuset Stockholm, Sweden.

Corresponding author: Pernilla Darlington, Department of Internal Medicine, Södersjukhuset, Stockholm, Sjukhusbacken 10, 118 83. Stockholm, e-mail address: pernilla.darlington@ki.se

Keywords: COVID-19, CT, quality of life, chest imaging

For peer review only - http://bmjopen.bmj.com/site/about/guidelines.xhtml

For peer review only

2023-05-09

ABSTRACT

Objective

We aimed to compare long-term outcomes in intensive care unit (ICU) survivors between the first and second/third waves of the COVID-19 pandemic. More specifically, to assess health-related quality of life (HRQL) and respiratory health 6 months post- ICU and to study potential associations between patient characteristic- and treatment variables regarding 6-month outcomes.

Design

Prospective cohort study.

Setting

Single-center study of adult COVID-19 patients with respiratory distress admitted to two Swedish ICUs during the first wave (1 March– 1 September 2020) and second/third waves (2 September 2020– 1 August 2021) with follow-up approximately 6 months after ICU discharge.

Participants

Critically ill COVID-19 patients who survived for at least 90 days.

Main outcome measures

HRQL, extent of residual changes on chest CT scan and pulmonary function were compared between the waves. General linear regression and multivariable logistic regression were used to present mean score differences (MSD) and odds ratios (OR) with 95% confidence intervals (CI).

For peer review only

2023-05-09

Results

Of the 456 (67%) critically ill COVID-19 patients who survived at least 90 days, 278 (61%) were included in the study. Six months after ICU discharge, HRQL was similar between survivors in the pandemic waves, except that the second/third wave survivors had better role physical (MSD 20.2, 95%CI: 7.3-33.1, p <0.01) and general health (MSD 7.2, 95%CI: 0.7-13.6, p = 0.03) and less bodily pain (MSD 12.2, 95%CI: 3.6-20.8, p<0.01), while first wave survivors had better diffusion capacity for carbon monoxide (DLCO) (OR 1.9, 95%CI: 1.1-3.5, p = 0.03).

Conclusions

This study indicates that even though intensive care treatment strategies have changed with time, there are few differences in long-term HRQL and respiratory health seems to remain at 6 months for patients surviving critical COVID-19 in the first and second/third waves of the pandemic.

Strengths and limitations of this study

*The main methodological strengths of the study lie in the prospective and comprehensive data collection of long-term outcomes in critically ill patients with COVID-19.

*The study included well-validated outcome tests and questionnaires.

* Sixty-one percent of the ICU survivors were included in the study, which may induce a risk of selection bias.

*Further survival rate and follow-up rate were higher for those from the first wave.

*Data on virus strains were not available, so analyses were not adjusted for this parameter.

For peer review only - http://bmjopen.bmj.com/site/about/guidelines.xhtml

2023-05-09

INTRODUCTION

Coronavirus disease 2019 (COVID-19), caused by severe acute respiratory syndrome coronavirus 2 (SARS-CoV-2), was declared a global pandemic in March 2020 and has resulted in numerous severely ill individuals in need of respiratory support[1] and millions of deaths worldwide[2]. The majority survived the acute episode of a critical illness but were confronted with physical and psychological sequelae after discharge from hospital which prolonged recovery[3]. Approximately 35-80% of the survivors experienced persistent symptoms of fatigue, dyspnea, joint pain, sleep disorders, and loss of memory and concentration lasting beyond 4 weeks after a moderate to severe acute infection, which is referred to as "post-acute COVID-19 syndrome"[4-6] Long-lasting respiratory complications as well as other functional deficits which may cause substantial morbidity have been reported among intensive care unit (ICU) survivors in the first year after hospital discharge[7-10]. Furthermore, a high number of residual abnormalities on chest CT scan have been found several months after discharge from hospital, and acute respiratory distress syndrome has been associated with more severe radiological findings[11]. Since the start of the pandemic the risk for hospital admission has varied as new variants of SARS-CoV-2 have emerged [12]. Likewise, worldwide different virus variants have been dominating the waves in Sweden [13]. Further, during the second and third waves, scientific and clinical understanding of the illness improved, and potentially more effective treatments were provided. Yet, whether functional outcomes have improved in line with the increase in knowledge about COVID-19 remains unexplored.

We aimed to compare long-term outcomes in ICU survivors between the first and second/third waves of the COVID-19 pandemic. More specifically, to assess health-related quality of life (HRQL) and respiratory health 6 months post- ICU. Moreover, to study

For peer review only - http://bmjopen.bmj.com/site/about/guidelines.xhtml

2023-05-09

potential associations between patient characteristic- and treatment variables regarding 6-month outcomes.

For peer review only

For peer review only - http://bmjopen.bmj.com/site/about/guidelines.xhtml

2023-05-09

METHODS

Study design and participants

This was a prospective, single-center cohort study of patients with acute COVID-19 infection admitted to two ICUs at Södersjukhuset, Stockholm, Sweden between 1 March 2020 and 1 August 2021. All patients who had been critically ill with a positive polymerase chain reaction test for COVID-19 and treated for respiratory failure with invasive ventilation, HFNO or non-invasive ventilation (NIV) in the ICU were eligible for inclusion in the study. Study approval was granted from the Swedish Ethical Review Authority (2020-03760, 2020-06106 and 2022-03531-02) and written informed consent was obtained from all included participants.

Setting

Before the pandemic, the two ICUs had a total number of 16 beds. During the first wave, the number of ICU beds expanded to a maximum of 60 beds in April and May 2020. At the beginning of the second wave, the number of ICU beds were 21, rising to 29 beds in November 2020 and further up to maximum of 33 beds in April and May 2021. At the end of the third wave, the number of beds were 21. The nurse:patient ratio was 1:1-2 and the physician:patient ratio 1:4-6 during all three waves, but the proportion of intensive care specialized nurses and physicians decreased during the peaks of the pandemic. None of the ICUs were exclusive for COVID-19 patients. The admission criteria to the ICU due to COVID-19 were need of respiratory support (i.e. HFNO/NIV/invasive ventilation). During second/third wave there was a step down for stable patients on high-flow treatment with

For peer review only - http://bmjopen.bmj.com/site/about/guidelines.xhtml

2023-05-09

oxygen (HFNO), but still under continues observation. Corticosteroids became standard treatment at the end of the first wave.

Data Collection

A detailed description of this data collection can be found in other publications [7, 8]. Data on demographic and clinical details were retrieved from medical charts regarding age (≤50, 50-65, >65 years), sex (male/female), body mass index (BMI), smoking habits (ever/never), comorbidities (i.e. diabetes, hypertension/cardiovascular disease, chronic lung disease), Simplified Acute Physiological Score III (SAPS 3; a scoring system used to predict mortality risk in the ICU, where a higher score indicates a higher mortality risk [14]), corticosteroid treatment (yes/no), respiratory support (invasive vs HFNO/NIV) and ICU length of stay.

Pandemic waves

The study was categorized into two time periods: the first wave (1 March–1 September 2020) and the second/third waves (2 September 2020–1 August 2021). In Sweden, the first cases of severely ill patients were admitted to the ICU at the beginning of March 2020 with a peak in April and then subsided during the summer [15]. Therefore, this time period has been considered to separate the end of the first wave [16]. The beginning of the second wave was estimated to start on 2 September 2020. Thereafter under a longer period, there was an increase in cases with one peak around new year and one peak in April 2021. There was no clear gap in time between the second and third waves, even if there was a clear decrease in

For peer review only - http://bmjopen.bmj.com/site/about/guidelines.xhtml

2023-05-09

cases in March 2021 [15], and the second and third waves were therefore grouped as one time period in this study.

Outcomes

The main outcomes were HRQL and respiratory health assessed approximately 6 months after ICU discharge.

HRQL was assessed with the self-administered RAND-36 Item Health Survey (RAND Corp), a version of The Medical Outcome Study (MOS) Short Form-36 (SF-36)[17]. SF-36 and RAND-36 are commonly used generic instrument for measuring HRQL and rather similar, except for some minor differences, e.g. RAND-36 lacks an authorized algorithm for calculation of Mental and Physical Component Summary scores [18].The Swedish translation (RAND-36 version 1.02) has been shown to be equivalent to the English[18]. It includes reference data for norm-based comparisons and has been shown to be an acceptable and reliable instrument of measuring HRQL in a Swedish general population [19]. The RAND-36 questionnaire includes the following eight domains: physical functioning (10 items), role physical (4 items), bodily pain (2 items), general health (5 items), vitality (4 items), social functioning (2 items), role emotional (3 items) and emotional well-being (5 items)[20]. Questionnaire responses were linearly transformed into scores between 0 and 100, where a higher score represents a higher HRQL[18, 20].

Residual changes were identified with a CT scan of the thoracic region using a Siemens Somatom Drive with a standard 120-kV CT thorax protocol as described in a previous publication[7] . International standard thoracic radiological terminology from the Fleischner Society[21] was used to identify the parenchymal changes related to SARS-CoV-2 infection,[22]

For peer review only - http://bmjopen.bmj.com/site/about/guidelines.xhtml

such as ground-glass opacities, subpleural bands, reticular pattern, and bronchiectasis. A semi-quantitative CT score (CT involvement score) was estimated based on lobar involvement and then calculated as a total score for all five lobes: 0: 0%, 1: <5%, 2: 5%–25%, 3: 26%–50%, 4: 51%–75%, 5: >75%, yielding a score ranging from 0 to 5 in each lobe and a global score of 0–25[23].

Pulmonary function was assessed as capacity for gas exchange, i.e., diffusion capacity for carbon monoxide (DLCO) (uncorrected value), according to standard methods[24]. Results were expressed in accordance with reference values (Hedenström)[25, 26] with DLCO <80% as cut-off for impaired pulmonary function[8, 9, 11, 27].

Statistical analyses

For comparison of HRQL, multivariable general linear regression models were used to estimate mean score differences (MSD) with 95% confidence intervals (CI). A 3–5-point differences between groups were regarded as minimally clinically important differences [28]. Missing items were accounted for with automatic calculation of the scores. If more than half of the scores in one domain were missing, no result was presented.

For respiratory health, multivariable logistic regression models were used with calculated odds ratios (OR) with 95% confidence intervals (CI). The analyses between first and second/third waves were adjusted for age (≤50, 50-65, >65 years), sex, smoking habits and chronic lung disease. These variables were chosen since they are known as risk factors for a reduced lung function [29]. Associations between patient- and treatment-related factors and outcomes were adjusted for age (≤65/>65 years), sex, comorbidity, corticosteroids and invasive ventilation, all known to influence the course of the disease in COVID-19 [30-32]. Two

For peer review only - http://bmjopen.bmj.com/site/about/guidelines.xhtml

2023-05-09

separate statistical analyses were conducted; one including participants with at least one test result and a second with participants who had complete data from all three examinations (chest CT scan, pulmonary function test and questionnaires). Statistical analyses were performed with Jamovi version 2.3.19 and an experienced biostatistician validated all analyses.

Patient and public involvement

Since the hospital was under heavy burden due to COVID-19 patients and members of public health were not involved in the set-up of the study.

For peer review only

2023-05-09

RESULTS

Participants

In total, 678 patients were treated for severe COVID-19 infection during the study period, among which 456 (67%) survived for at least 90 days after ICU discharge. Of these, 278 (61%) were followed-up 6 months after ICU discharge (Figure 1). There were 182 (72%) who survived from the first wave and 122 (67%) had a follow-up. For the second/third waves 274 (64%) survived and 156 (57%) had a follow-up. First-wave survivors were younger, had lower BMI, fewer cardiovascular diseases, higher CRP max, and were more often treated with invasive ventilation, but less frequently with corticosteroids (Table 1). Among survivors with chronic lung disease, the vast majority had asthma. Patient characteristics and ICU-related data were similar between survivors who attended the follow-up and non-participants (Supplementary Table 1), but among non-responders who declined follow-up, non-invasive ventilation was more common (Supplementary Table 2). Further, distribution of age, sex, comorbidities, corticosteroid treatment and type of ventilation were similar between participants who completed the questionnaires and performed the pulmonary function test and chest CT scan and those who did not (Supplementary Table 3).

For peer review only - http://bmjopen.bmj.com/site/about/guidelines.xhtml

For peer review only

2023-05-09

Table 1. Characteristics of included COVID-19 intensive care unit survivors across the first and second/third pandemic waves.

	**First wave** (1 March–1 Sept 2020)	**Second/third waves** (2 Sept 2020 – 1 Aug 2021)	**p-value**
Total number, n	122	156	
Follow-up time, months, Md (IQR)	5 (4-5)	6 (5-8)	<0.001*
Age, years Md (IQR)	58 (50-66)	61 (53-69)	<0.05*
Male n (%)	93 (76)	110 (71)	0.34
BMI, kg/m^2 Md (IQR)	28 (25-31)	29 (26-33)	<0.05*
Diabetes n (%)	24 (20)	36 (23)	0.56
Hypertension n (%)	53 (43)	72 (46)	0.72
Cardiovascular disease n (%)	12 (10)	30 (19)	<0.05*
Chronic lung disease n (%)	22 (18)	37 (24)	0.30
Ever smoker n (%)	46 (38)	74 (47)	0.11
CRP max, mg/L, Md (IQR)	310 (199-355)	182 (126-248)	<0.001*
SAPS 3, Md (IQR)	54 (50-60)	56 (52-59)	0.52
Invasive ventilation n (%)	72 (59)	64 (41)	<0.01*
Corticosteroid treatment n (%)	49 (40)	156 (100)	<0.001*
Length of ICU stay (days), Md (IQR)	13 (4-23)	9 (6-16)	0.33

BMI= body mass index; CRP = C reactive protein; IQR=Interquartile range; Md=Median; SAPS= Simplified Acute Physiology Score (missing information in 37 participants); *p value <0.05.

For peer review only

HRQL

Among the 187 ICU survivors who reported HRQL data, 76 were treated in the ICU for COVID-19 during the first wave. First-wave survivors reported lower scores in all HRQL domains, with clinically relevant and statistically significant differences in the role physical domain (MSD 20.2, 95%CI: 7.3 to 33.1, p<0.01), bodily pain (MSD 12.2, 95%CI: 3.6 to 20.8, p <0.01) and general health (MSD 7.2, 95%CI: 0.7 to 13.6, p = 0.03) (Table 2).

For peer review only - http://bmjopen.bmj.com/site/about/guidelines.xhtml

2023-05-09

Table 2. Health-related quality of life (HRQL) comparing COVID-19 intensive care unit survivors across the first and second/third waves, presented as mean score differences (MSD) with 95% confidence intervals (CI), respectively Odds ratios (OR) with 95% confidence intervals (CI) of CT score and diffusion capacity (DLCO). First wave patients' data were used as a reference.

Health-related quality of life domains	**First wave**<br>Mean scores (95%CI)		**Second/third waves**<br>Mean scores (95%CI)		**Unadjusted analysis**<br>Mean score differences (95%CI)<br>p-value	**Adjusted analysis**<br>Mean score differences (95%CI)<br>p-value
Total number	76		111			
Physical function	62<br>(57-68)		64<br>(58-69)		1.3<br>(-6.7 to 9.3)<br>0.75	3.6<br>(-4.2 to 11.5)<br>0.36
Role physical	31<br>(22-40)		50<br>(41-58)		18.5*<br>(5.6 to 31.3)<br><0.01	20.2*<br>(7.3-33.1)<br><0.01
Bodily pain	59<br>(52-66)		69<br>(64-75)		10.8*<br>(2.3 to 19.3)<br>0.01	12.2*<br>(3.6 to 20.8)<br><0.01
General health	49<br>(44-54)		56<br>(51-60)		6.3<br>(-0.2 to 12.8)<br>0.06	7.2*<br>(0.7 to 13.6)<br>0.03
Vitality	47<br>(41-52)		53<br>(48-58)		6.3<br>(-0.9 to 13.5)<br>0.09	7.0<br>(-0.2 to 14.2)<br>0.06
Social function	62<br>(56-68)		67<br>(62-73)		5.6<br>(-2.9 to 14.0)<br>0.19	6.3<br>(-2.2 to 14.7)<br>0.14
Role emotional	58<br>(48-68)		68<br>(60-75)		9.9<br>(-2.6 to 22.5)<br>0.12	12.0<br>(-0.5 to 24.6)<br>0.06
Mental health	67<br>(62-72)		74<br>(70-78)		6.8*<br>(0.8 to 12.8)<br>0.03	6.9<br>(-0.8 to 13.1)<br>0.03
Outcomes	**First wave**		**Second/third waves**		OR (95%CI)<br>p-value	OR (95%CI)<br>p-value
	Numbers	Median (IQR)	Numbers	Median (IQR)		
CT score<br>0<br>≥1	 25<br>65	 0<br>7 (5-10)	 21<br>101	 0<br>9 (5-13)	 Reference 1.0<br>1.8 (1.0-3.6)<br>0.07	 Reference 1.0<br>1.8 (0.9-3.7)<br>0.10
DLCO % of predicted<br>≥80<br><80	 64<br>36	 96 (90-109)<br>68 (62-75)	 49<br>57	 91 (86-96)<br>65 (53-71)	 Reference 1.0<br>2.1 (1.2-3.6)*<br>0.01	 Reference 1.0<br>1.9 (1.1-3.5)*<br>0.03

For peer review only

For peer review only - http://bmjopen.bmj.com/site/about/guidelines.xhtml

2023-05-09

Abbreviations: DLCO=diffusion capacity for carbon monoxide; CT=computed tomography; IQR = interquartile range. The models were adjusted for age, sex, tobacco smoking, chronic lung disease. *p-value <0.05.

Similar results were found for survivors of COVID-19 with complete case data, except for non-significant results for general health (MSD 6.9, 95%CI: -1.3 to 15.2, p = 0.1). In the multivariate analysis, older age (MSD -12.9, 95%CI: -21.0 to -4.8, p<0.01) was associated with worse physical function and similar was seen for those with diabetes (MSD -11.0, 95%CI: -21.0 to -1.1, p = 0.03). Diabetes also associated with lower scores on general health (MSD -11.6, 95%CI: -20.0 to -3.3, p<0.01) and mental health (MSD -9.5, 95%CI: -17.3 to -1.7, p = 0.02). Survivors treated with invasive ventilation were more likely to report lower scores on bodily pain (MSD -11.8, 95%CI: -20.2 to -3.4, p<0.01). Lower scores on role emotional were seen for those with chronic lung disease (MSD -24.2, 95%CI: -39.7 to -8.8, p<0.01) (Table 3). The univariate analyses can be found in Supplementary Table 4. Similar results were seen in the complete case analysis (i.e participants who had data from all three examinations with questionnaires, chest CT-scan and pulmonary function), where also diabetes associated with more bodily pain (MSD -16.4, 95%CI: -29.5 to -3.3, p = 0.02) but not with lower scores for mental health (MSD -8.9, 95%CI: -18.8 to 1.0, p = 0.08).

For peer review only

2023-05-09

Table 3. Multivariate analysis of patient- and clinical characteristics in relation to health- related quality of life (HRQL) in COVID-19 intensive care unit survivors presented as adjusted mean score differences (MSD) with 95% confidence intervals (CI).

**Variables**	**Physical function**<br>MSD (95%CI)<br>p-value	**Role physical**<br>MSD (95%CI)<br>p-value	**Bodily pain**<br>MSD (95%CI)<br>p-value	**General health**<br>MSD (95%CI)<br>p-value	**Vitality**<br>MSD (95%CI)<br>p-value	**Social function**<br>MSD (95%CI)<br>p-value	**Role emotional**<br>MSD (95%CI)<br>p-value	**Mental health**<br>MSD (95%CI)<br>p-value
**Age, years**<br>≤65<br>>65	Reference 1.0<br>-12.9*<br>(-21.0 to -4.8)<br><0.01	Reference 1.0<br>-1.5<br>(-15.0 to 12.0)<br>0.83	Reference 1.0<br>-5.3<br>(-14.0 to 3.5)<br>0.24	Reference 1.0<br>-2.2<br>(-9.0 to 4.7)<br>0.54	Reference 1.0<br>2.4<br>(-5.3 to 10.0)<br>0.54	Reference 1.0<br>-2.6<br>(-11.6 to 6.3)<br>0.56	Reference 1.4<br>1.7<br>(-11.8 to 14.7)<br>0.83	Reference 1.0<br>1.5<br>(-4.8 to 7.9)<br>0.64
**Sex**<br>Female<br>Male	Reference 1.0<br>5.7<br>(-3.2 to 14.6)<br>0.21	Reference 1.0<br>11.1<br>(-3.6 to 25.8)<br>0.14	Reference 1.0<br>4.7<br>(-4.8 to 14.2)<br>0.33	Reference 1.0<br>4.2<br>(-3.2 to 11.6)<br>0.26	Reference 1.0<br>6.6<br>(-1.7 to 15.0)<br>0.12	Reference 1.0<br>9.8<br>(-0.1 to 19.5)<br>0.05	Reference 1.0<br>-4.2<br>(-18.6 to 10.2)<br>0.56	Reference 1.0<br>1.4<br>(-5.5 to 8.4)<br>0.69
**Diabetes**<br>No<br>Yes	Reference 1.0<br>-11.0*<br>(-21.0 to -1.1)<br>0.03	Reference 1.0<br>-8.5<br>(-25.1 to 8.1)<br>0.31	Reference 1.0<br>-9.2<br>(-19.9 to 1.5)<br>0.09	Reference 1.0<br>-11.6*<br>(-20.0 to -3.3)<br><0.01	Reference 1.0<br>-6.3<br>(-13.2 to 9.9)<br>0.18	Reference 1.0<br>-5.6<br>(-16.5 to 5.3)<br>0.31	Reference 1.0<br>-11.5<br>(-27.6 to 4.6)<br>0.16	Reference 1.0<br>-9.5*<br>(-17.3 to -1.7)<br>0.02
**Hypertension/ cardiovascular disease**<br>No<br>Yes	Reference 1.0<br>-3.2<br>(-11.5 to 5.3)<br>0.46	Reference 1.0<br>-1.1<br>(-15.1 to 13.0)<br>0.88	Reference 1.0<br>-6.4<br>(-15.5 to 2.6)<br>0.16	Reference 1.0<br>2.4<br>(-4.7 to 9.4)<br>0.51	Reference 1.0<br>1.7<br>(-6.2 to 9.6)<br>0.67	Reference 1.0<br>1.1<br>(-8.1 to 10.4)<br>0.81	Reference 1.0<br>1.2<br>(-12.6 to 14.9)<br>0.87	Reference 1.0<br>3.4<br>(-3.3 to 1.0)<br>0.32
**Chronic lung disease**<br>No<br>Yes	Reference 1.0<br>-5.8<br>(-15.5 to 3.8)<br>0.24	Reference 1.0<br>-13.6<br>(-29.4 to 2.2)<br>0.09	Reference 1.0<br>-5.6<br>(-15.9 to 4.6)<br>0.28	Reference 1.0<br>-6.9<br>(-14.9 to 1.1)<br>0.09	Reference 1.0<br>-8.3<br>(-17.3 to 0.7)<br>0.07	Reference 1.0<br>-2.9<br>(-13.4 to 7.4)<br>0.57	Reference 1.0<br>-24.2*<br>(-39.7 to -8.8)<br><0.01	Reference 1.0<br>-3.6<br>(-11.0 to 3.9)<br>0.35
**Ventilation support**<br>HFNO/NIV<br>invasive ventilation	Reference 1.0<br>-6.7<br>(-14.6 to 1.1)<br>0.09	Reference 1.0<br>-8.5<br>(-20.6 to 5.4)<br>0.2	Reference 1.0<br>-11.8*<br>(-20.2 to -3.4)<br><0.01	Reference 1.0<br>-1.6<br>(-8.2 to 4.9)<br>0.63	Reference 1.0<br>0.4<br>(-7.7 to 7.0)<br>0.93	Reference 1.0<br>-7.5<br>(-16.1 to 1.0)<br>0.08	Reference 1.0<br>0.5<br>(-12.2 to 13.2)<br>0.94	Reference 1.0<br>3.3<br>(-2.9 to 9.4)<br>0.30
**In-ICU corticosteroids**<br>No<br>Yes	Reference 1.0<br>-6.1<br>(-18.3 to 6.1)<br>0.33	Reference 1.0<br>-11.9<br>(-32.4 to 8.5)<br>0.25	Reference 1.0<br>-4.5<br>(-17.7 to 8.8)<br>0.51	Reference 1.0<br>-4.6<br>(-14.9 to 5.7)<br>0.38	Reference 1.0<br>-1.7<br>(-13.2 to 9.9)<br>0.77	Reference 1.0<br>-0.6<br>(-14.1 to 12.9)<br>0.93	Reference 1.0<br>3.6<br>(-16.4 to 23.5)<br>0.72	Reference 1.0<br>1.3<br>(-8.3 to 10.9)<br>0.79

Abbreviations: HFNO=high flow nasal oxygen; NIV=non-invasive ventilation; All the variables in the univariate analysis were included in the multivariate analysis with adjustment for time during the pandemic. *Statistically significant p-value <0.05.

Pulmonary outcomes

Compared with first wave survivors, patients in the second/third waves had worse pulmonary function with lower DLCO (OR 1.9, CI: 1.1-3.5, p = 0.03) (Table 2). Similar findings were seen in patients who had complete data from the three examinations.

For peer review only

For peer review only - http://bmjopen.bmj.com/site/about/guidelines.xhtml

Factors associated with worse radiological pulmonary outcome (i.e., CT score≥1) were older age (>65 years) (OR 4.5, CI: 1.7-11.8, p<0.01), male sex (OR 2.8, CI: 1.3-6.3, p = 0.01) and ICU corticosteroid treatment (OR 4.8, CI: 1.3-17.0, p = 0.02) (Table 4).

Table 4. Patient- and clinical characteristics in relation to pulmonary outcomes in COVID-19 intensive care unit survivors presented as odds ratio (OR) with 95% confidence intervals (CI).

**Variables**	**CT score≥1**			**DLCO <80%**		
	Numbers	Univariate OR (95%CI) p-value	Multivariate OR (95%CI) p-value	Numbers	Univariate OR (95%CI) p-value	Multivariate OR (95%CI) p-value
**Age, years**						
≤65	141	Reference 1.0	Reference 1.0	133	Reference 1.0	Reference 1.0
>65	71	4.3 (1.8-10.7)* <0.01	4.5 (1.7-11.8)* <0.01	73	2.2 (1.2-3.9)* <0.01	1.9 (1.0-3.6)* 0.04
**Sex**						
Female	61	Reference 1.0	Reference 1.0	57	Reference 1.0	Reference 1.0
Male	151	1.8 (0.9-3.6) 0.08	2.8 (1.3-6.3)* 0.01	149	0.5 (0.3-0.9)* 0.02	0.4 (0.2-0.9)* 0.02
**Diabetes**						
No	166	Reference 1.0	Reference 1.0	141	Reference 1.0	Reference 1.0
Yes	46	1.2 (0.5-2.7) 0.69	0.8 (0.3-2.0) 0.65	65	1.7 (0.9-3.5) 0.12	1.5 (0.7-3.3) 0.27
**Hypertension/ cardiovascular disease**						
No	108	Reference 1.0	Reference 1.0	103	Reference 1.0	Reference 1.0
Yes	104	1.4 (0.7-2.7) 0.13	1.2 (0.5-2.5) 0.72	103	1.8 (1.0-3.2)* 0.04	1.5 (0.8-2.9) 0.18
**Chronic lung disease**						
No	163	Reference 1.0	Reference 1.0	162	Reference 1.0	Reference 1.0
Yes	49	1.1 (0.5-2.4) 0.80	1.6 (0.7-4.0) 0.30	44	1.1 (0.6-2.2) 0.70	0.8 (0.4-1.8) 0.69
**Ventilation support**						
HFNO/NIV	105	Reference 1.0	Reference 1.0	102	Reference 1.0	Reference 1.0
invasive ventilation	107	1.4 (0.7-2.8) 0.28	1.4 (0.7-3.0) 0.32	104	1.2 (0.7-2.0) 0.57	1.3 (0.7-2.5) 0.35
**In-ICU corticosteroids**						
No	57	Reference 1.0	Reference 1.0	58	Reference 1.0	Reference 1.0
Yes	155	3.0 (1.5-6.0)* <0.01	4.8 (1.3-17.0)* 0.02	148	2.5 (1.3-4.9)* <0.01	1.7 (0.7-4.2) 0.21

Abbreviations: DLCO=diffusion capacity for carbon monoxide; HFNO=high flow nasal oxygen; NIV=non-invasive ventilation; multivariate analysis included all the variables in the univariate analysis and was adjusted for time during the pandemic. *Statistically significant p-value <0.05.

For peer review only

For peer review only - http://bmjopen.bmj.com/site/about/guidelines.xhtml

For the complete case analysis, only older age was associated with worse CT scores (OR 3.3, 95%CI: 1.1 to 10.1, p = 0.04).

Worse pulmonary function (DLCO<80%) was associated with older age (OR 1.9, CI: 1.0-3.6, p = 0.04) and female sex (with female as reference) (OR 0.4, CI: 0.2-0.9, p = 0.02) (Table 4), but in the complete case analysis only female sex was associated with worse capacity for gas-exchange (OR 0.3, 95%CI: 0.1 to 0.8, p = 0.02).

For peer review only

DISCUSSION

In this prospective cohort study of COVID-19 ICU survivors, HRQL and pulmonary outcomes were relatively similar between the pandemic waves, despite a changing patient case-mix, new virus variants and changes in intensive care treatment strategies, except that first wave survivors had better pulmonary function, measured with the capacity for gas-exchange (DLCO) and second/third wave survivors reported better role physical, general health and had less bodily pain.

In our study cohort ICU-survivors treated early in the pandemic seemed to have more severe disease since they were younger with a longer stay in the ICU and to a higher extent treated with invasive ventilation. These differences in severity of disease may be due to viral strain [33] and that later in the pandemic corticosteroid treatment was routinely given [34]. The majority were unvaccinated in Sweden during the time for study inclusion [35]. The ICU-survivors later in the pandemic seemed to be more fragile because of older age, higher BMI and cardiovascular disease. Most evidence indicates a small reduction in mortality rates between the first and second/third pandemic waves among critically ill patients[36, 37]. However, data about mortality trends during the pandemic vary depending on study time frames, outcomes, case-mix and sample size[2]. Only a few studies have investigated whether long-term morbidity follows the mortality results. One of these studies was a multinational study, in which 1000 patients with COVID-19 and severe hypoxaemia were randomized to either 6 mg or 12 mg dexamethasone, but no differences between groups were found in 3-months mortality or HRQL[38].

A systematic review and meta-analysis showed that those with more severe initial illness had poorer long-term recovery after COVID-19 infection[39]. In our study, risk factors for reduced quality of life in at least one parameter at follow-up in ICU survivors treated because of

For peer review only - http://bmjopen.bmj.com/site/about/guidelines.xhtml

COVID-19 were older age, diabetes, chronic lung disease and invasive ventilation. Diabetes as a risk factor for critical disease and mortality was identified early in the pandemic [40]. Age as a risk factor for severe disease and worse radiological outcome is well-known and has been described previously [9, 41]. In line with this, severity of disease has in previous literature been reported to be associated with more remaining health related symptoms at follow-up [9]. As previously reported about the first wave survivors in the cohort, their quality-of-life was reduced compared with the general Swedish population [8], also consistent with data from other populations [31].

For pulmonary healing, older age and corticosteroid treatment were associated with more residual changes on chest CT scan. Regarding treatment with corticosteroids there are still little known about possible harm and long-term effects in COVID-19 [36, 42].

Increasing age and female sex were associated with worse lung function at follow-up. In general, there was a predominance of males who become critically ill[43]. The females in need of ICU treatment in our cohort may have had a worse pulmonary impairment before COVID-19 because there was a discrepancy between CT score and the capacity for gas-exchange (DLCO) in females. An association between female sex and worse DLCO values in hospitalized patients has also been reported by others[27].

Strength and limitations

The main methodological strengths of the study lie in the prospective and comprehensive data collection together with the use of well-validated outcome tests and questionnaires. The study also has a number of limitations. Firstly, number of missing for follow-up due to

For peer review only - http://bmjopen.bmj.com/site/about/guidelines.xhtml

administrative reasons (n=67) may contribute to increase the risk of selection bias. However, it is likely to believe that those patients were missed at random and not systematically. During this time, the hospital was heavily burdened of COVID-19 and all nurses and physicians were needed in direct patient care and eligible patients were sometimes identified after ICU discharge.

Of those who declined follow-up, fewer needed invasive ventilation and they might have reached complete recovery and considered follow-up redundant. Though, when comparing baseline data for all survivors, no statistically significant differences were found between groups. Further, the results may be biased by the higher survival rate in the first wave which may be explained by changes in patient-mix, viral strain and differences in treatment strategies, and the follow-up rate was higher for those in the first wave. There are also unmeasured possible residual confounders such as experience, bed occupancy and staff. The lack of baseline data prevents identification of new-onset problems. Yet, data on pulmonary function and HRQL is difficult to obtain pre-ICU. Further, the HRQL results were self-reported and may include a risk of response shift. Since HRQL aspects include many variables, and several analyses were conducted, there is also a risk that some statistically significant results may be due to chance.

For peer review only

The knowledge about long-term outcomes after critical disease of COVID-19 may also be applicable for other causes of acute respiratory distress syndrome (ARDS) as well as treatment in the ICU of various conditions where, consistent with our findings, functional impairments are common [44, 45]. In addition, to reduce morbidity in ICU patients with COVID-19 infection it is of great importance to investigate what factors may negatively affect recovery.

For peer review only - http://bmjopen.bmj.com/site/about/guidelines.xhtml

CONCLUSION

This study suggests that even though patient case-mix has changed, new variants of SARS-CoV-2 have emerged, and intensive care treatment strategies have changed since the start of the pandemic, long-term HRQL and impaired respiratory health remains similar for COVID-19 patients with critical illness. Further, factors associated with severity of disease may play a role for the recovery.

For peer review only

For peer review only - http://bmjopen.bmj.com/site/about/guidelines.xhtml

DECLARATIONS

Ethics approval and consent to participate

Study approval was granted from the Swedish Ethical Review Authority (Diary number: 2020-03760) and written informed consent was obtained from all included participants.

Declaration of interests

The authors declare that they have no competing interests.

Availability of data and materials

The datasets used and/or analyzed during the current study are available from the corresponding author upon reasonable request.

Funding

Departmental funding only.

Author contributions

PD and MR contributed to conceptualisation, methodology, formal analysis, investigation, writing – original draft. MC contributed to methodology, investigation, writing – review and editing. GH contributed to methodology, investigation, writing – review and editing. AH: conceptualisation, investigation, writing – review and editing. EJA contributed to conceptualisation, formal analysis, writing – review and editing. AS contributed to conceptualisation, methodology, investigation, formal analysis, writing draft – review and editing. All authors read and approved the final manuscript.

For peer review only

Acknowledgements

We would like to thank all patients who participated in the study and caregivers involved in the follow-up.

For peer review only

For peer review only - http://bmjopen.bmj.com/site/about/guidelines.xhtml

REFERENCES

1. Lim ZJ, Subramaniam A, Ponnapa Reddy M, et al. Case Fatality Rates for Patients with COVID-19 Requiring Invasive Mechanical Ventilation. A Meta-analysis. *Am J Respir Crit Care Med*. 2021; 203: 54-66.
2. Fan G, Yang Z, Lin Q, Zhao S, Yang L and He D. Decreased Case Fatality Rate of COVID-19 in the Second Wave: A study in 53 countries or regions. *Transbound Emerg Dis*. 2021; 68: 213-5.
3. Evans RA, McAuley H, Harrison EM, et al. Physical, cognitive, and mental health impacts of COVID-19 after hospitalisation (PHOSP-COVID): a UK multicentre, prospective cohort study. *Lancet Respir Med*. 2021; 9: 1275-87.
4. Chopra V, Flanders SA, O'Malley M, Malani AN and Prescott HC. Sixty-Day Outcomes Among Patients Hospitalized With COVID-19. *Ann Intern Med*. 2021; 174: 576-8.
5. Carfi A, Bernabei R, Landi F and Gemelli Against C-P-ACSG. Persistent Symptoms in Patients After Acute COVID-19. *JAMA*. 2020; 324: 603-5.
6. Garrigues E, Janvier P, Kherabi Y, et al. Post-discharge persistent symptoms and health-related quality of life after hospitalization for COVID-19. *J Infect*. 2020; 81: e4-e6.
7. Hanna G, Bankler S, Schandl A, et al. The role of ventilatory support for long-term outcomes after critical infection with COVID-19: A prospective cohort study. *Clin Respir J*. 2022; 16: 63-71.
8. Schandl A, Hedman A, Lynga P, et al. Long-term consequences in critically ill COVID-19 patients: A prospective cohort study. *Acta Anaesthesiol Scand*. 2021; 65: 1285-92.
9. Huang C, Huang L, Wang Y, et al. 6-month consequences of COVID-19 in patients discharged from hospital: a cohort study. *Lancet*. 2021; 397: 220-32.
10. Jacobson KB, Rao M, Bonilla H, et al. Patients With Uncomplicated Coronavirus Disease 2019 (COVID-19) Have Long-Term Persistent Symptoms and Functional Impairment Similar to Patients with Severe COVID-19: A Cautionary Tale During a Global Pandemic. *Clin Infect Dis*. 2021; 73: e826-e9.
11. Han X, Fan Y, Alwalid O, et al. Six-month Follow-up Chest CT Findings after Severe COVID-19 Pneumonia. *Radiology*. 2021; 299: E177-E86.
12. Twohig KA, Nyberg T, Zaidi A, et al. Hospital admission and emergency care attendance risk for SARS-CoV-2 delta (B.1.617.2) compared with alpha (B.1.1.7) variants of concern: a cohort study. *Lancet Infect Dis*. 2022; 22: 35-42.
13. Strålin K, Bruce D, Wahlström E, et al. Impact of the Alpha VOC on disease severity in SARS-CoV-2-positive adults in Sweden. *J Infect*. 2022; 84: e3-e5.
14. Metnitz PG, Moreno RP, Almeida E, et al. SAPS 3--From evaluation of the patient to evaluation of the intensive care unit. Part 1: Objectives, methods and cohort description. *Intensive Care Med*. 2005; 31: 1336-44.
15. Ludvigsson JF. How Sweden approached the COVID-19 pandemic: Summary and commentary on the National Commission Inquiry. *Acta Paediatr*. 2023; 112: 19-33.
16. Zhang SX, Arroyo Marioli F, Gao R and Wang S. A Second Wave? What Do People Mean by COVID Waves? - A Working Definition of Epidemic Waves. *Risk Manag Healthc Policy*. 2021; 14: 3775-82.
17. Ware JE and Sherbourne CD. The MOS 36-item short-form health survey (SF-36). I. Conceptual framework and item selection. *Med Care*. 1992; 30: 473-83.
18. Orwelius L, Nilsson M, Nilsson E, et al. The Swedish RAND-36 Health Survey - reliability and responsiveness assessed in patient populations using Svensson's method for paired ordinal data. *J Patient Rep Outcomes*. 2017; 2: 4.
19. Ohlsson-Nevo E, Hiyoshi A, Noren P, Moller M and Karlsson J. The Swedish RAND-36: psychometric characteristics and reference data from the Mid-Swed Health Survey. *J Patient Rep Outcomes*. 2021; 5: 66.
20. Hays RD, Sherbourne CD and Mazel RM. The RAND 36-Item Health Survey 1.0. *Health Econ*. 1993; 2: 217-27.

For peer review only - http://bmjopen.bmj.com/site/about/guidelines.xhtml

21. Hansell DM, Bankier AA, MacMahon H, McLoud TC, Muller NL and Remy J. Fleischner Society: glossary of terms for thoracic imaging. *Radiology*. 2008; 246: 697-722.
22. Chung M, Bernheim A, Mei X, et al. CT Imaging Features of 2019 Novel Coronavirus (2019-nCoV). *Radiology*. 2020; 295: 202-7.
23. Francone M, Iafrate F, Masci GM, et al. Chest CT score in COVID-19 patients: correlation with disease severity and short-term prognosis. *Eur Radiol*. 2020; 30: 6808-17.
24. Graham BL, Brusasco V, Burgos F, et al. 2017 ERS/ATS standards for single-breath carbon monoxide uptake in the lung. *Eur Respir J*. 2017; 49.
25. Hedenstrom H, Malmberg P and Fridriksson HV. Reference values for lung function tests in men: regression equations with smoking variables. *Ups J Med Sci*. 1986; 91: 299-310.
26. Hedenstrom H, Malmberg P and Agarwal K. Reference values for lung function tests in females. Regression equations with smoking variables. *Bull Eur Physiopathol Respir*. 1985; 21: 551-7.
27. Mendez R, Latorre A, Gonzalez-Jimenez P, et al. Reduced Diffusion Capacity in COVID-19 Survivors. *Ann Am Thorac Soc*. 2021; 18: 1253-5.
28. Samsa G, Edelman D, Rothman ML, Williams GR, Lipscomb J and Matchar D. Determining clinically important differences in health status measures: a general approach with illustration to the Health Utilities Index Mark II. *Pharmacoeconomics*. 1999; 15: 141-55.
29. Raherison C and Girodet PO. Epidemiology of COPD. *Eur Respir Rev*. 2009; 18: 213-21.
30. Dessie ZG and Zewotir T. Mortality-related risk factors of COVID-19: a systematic review and meta-analysis of 42 studies and 423,117 patients. *BMC Infect Dis*. 2021; 21: 855.
31. Rosa RG, Cavalcanti AB, Azevedo LCP, et al. Association between acute disease severity and one-year quality of life among post-hospitalisation COVID-19 patients: Coalition VII prospective cohort study. *Intensive Care Med*. 2023; 49: 166-77.
32. Horby P, Lim WS, Emberson JR, et al. Dexamethasone in Hospitalized Patients with Covid-19. *N Engl J Med*. 2021; 384: 693-704.
33. Pavan M, Bassani D, Sturlese M and Moro S. From the Wuhan-Hu-1 strain to the XD and XE variants: is targeting the SARS-CoV-2 spike protein still a pharmaceutically relevant option against COVID-19? *J Enzyme Inhib Med Chem*. 2022; 37: 1704-14.
34. Wagner C, Griesel M, Mikolajewska A, et al. Systemic corticosteroids for the treatment of COVID-19. *Cochrane Database Syst Rev*. 2021; 8: CD014963.
35. Isitt C, Sjöholm D, Hergens MP, Granath F and Nauclér P. The early impact of vaccination against SARS-CoV-2 in Region Stockholm, Sweden. *Vaccine*. 2022; 40: 2823-7.
36. Carbonell R, Urgeles S, Rodriguez A, et al. Mortality comparison between the first and second/third waves among 3,795 critical COVID-19 patients with pneumonia admitted to the ICU: A multicentre retrospective cohort study. *Lancet Reg Health Eur*. 2021; 11: 100243.
37. Dongelmans DA, Termorshuizen F, Brinkman S, et al. Characteristics and outcome of COVID-19 patients admitted to the ICU: a nationwide cohort study on the comparison between the first and the consecutive upsurges of the second wave of the COVID-19 pandemic in the Netherlands. *Ann Intensive Care*. 2022; 12: 5.
38. Granholm A, Kjaer MN, Munch MW, et al. Long-term outcomes of dexamethasone 12 mg versus 6 mg in patients with COVID-19 and severe hypoxaemia. *Intensive Care Med*. 2022; 48: 580-9.
39. Han Q, Zheng B, Daines L and Sheikh A. Long-Term Sequelae of COVID-19: A Systematic Review and Meta-Analysis of One-Year Follow-Up Studies on Post-COVID Symptoms. *Pathogens*. 2022; 11.
40. Huang I, Lim MA and Pranata R. Diabetes mellitus is associated with increased mortality and severity of disease in COVID-19 pneumonia - A systematic review, meta-analysis, and meta-regression. *Diabetes Metab Syndr*. 2020; 14: 395-403.
41. Chen Y, Ding C, Yu L, et al. One-year follow-up of chest CT findings in patients after SARS-CoV-2 infection. *BMC Med*. 2021; 19: 191.

For peer review only - http://bmjopen.bmj.com/site/about/guidelines.xhtml

42. Moreno G, Carbonell R, Martin-Loeches I, et al. Corticosteroid treatment and mortality in mechanically ventilated COVID-19-associated acute respiratory distress syndrome (ARDS) patients: a multicentre cohort study. *Ann Intensive Care*. 2021; 11: 159.
43. Peckham H, de Gruijter NM, Raine C, et al. Male sex identified by global COVID-19 meta-analysis as a risk factor for death and ITU admission. *Nat Commun*. 2020; 11: 6317.
44. Herridge MS, Tansey CM, Matté A, et al. Functional disability 5 years after acute respiratory distress syndrome. *N Engl J Med*. 2011; 364: 1293-304.
45. Hofhuis JG, van Stel HF, Schrijvers AJ, Rommes JH and Spronk PE. ICU survivors show no decline in health-related quality of life after 5 years. *Intensive Care Med*. 2015; 41: 495-504.

For peer review only

For peer review only - http://bmjopen.bmj.com/site/about/guidelines.xhtml

Figure legend

Figure 1. Flowchart of study inclusion.

For peer review only

For peer review only - http://bmjopen.bmj.com/site/about/guidelines.xhtml

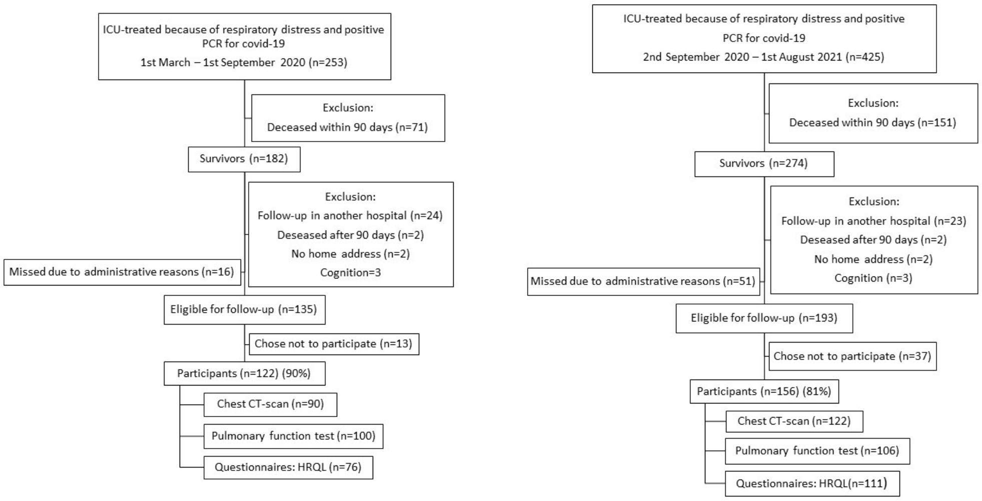

Flowchart of study inclusion.

108x60mm (300 x 300 DPI)

SUPPLEMENTARY FILE

Supplementary Table 1. Comparisons of patient characteristics between participants and non-participants among those invited for follow-up.

Characteristics	Participants	Non-participants	p-values	Missing
Total number, n (%)	278 (85)	50 (15)		
Age, years mean±SD	59.5± 11.9	62.0± 13.7	0.18	
Sex, n (%)<br>Male	203 (73)	37 (74)	0.56	
SAPS 3 score, mean±SD	55.6± 7.8	55.8± 7.6	0.90	51
Ventilator treatment, n (%)<br>Invasive	136 (49)	15 (30)	<0.05	

Student's t-test and Chi_2-test were used where appropriate. Statistically significant p-value <0.05.

Supplementary Table 2. Comparisons of patient characteristics between participants and non-participants including all survivors >3 months.

Characteristics	Participants	Non-participants	p-values	Missing
Total number, n (%)	278 (61)	178 (39)		
Age, years mean±SD	59.5± 11.9	61.1± 13.4	0.19	
Sex, n (%)<br>Male	203 (73)	128 (72)	0.83	
SAPS 3 score, mean±SD	55.6± 7.8	55.7± 7.6	0.95	82
Ventilator treatment, n (%)<br>Invasive	136 (49)	89 (50)	0.85	

Student's t-test and Chi_2-test were used where appropriate. Statistically significant p-value <0.05.

For peer review only

Supplementary Table 3. Comparison between participants who completed the questionnaires; performed the pulmonary function test; chest CT scan and those who did not.

Measure	Characteristic	Responder	Non-responder	p-value
**RAND-36**		N=187	N=91	
	Age ≥ 65	66 (36)	27 (29)	0.35
	Male	134 (72)	69 (76)	0.46
	Diabetes	39 (21)	21 (23)	0.67
	Hypertension/ cardiovascular disease	99 (53)	40 (44)	0.16
	Chronic lung disease	43 (23)	16 (18)	0.30
	Mechanical ventilation	91 (49)	45 (50)	0.90
	Corticosteroid treatment	143 (76)	62 (68)	0.14
**Pulmonary function test**		N=206	N=72	
	Age ≥ 65	73 (35)	20 (28)	0.24
	Male	149 (72)	54 (75)	0.67
	Diabetes	41 (20)	19 (26)	0.25
	Hypertension/ cardiovascular disease	103 (50)	36 (50)	1.00
	Chronic lung disease	44 (21)	15 (21)	0.92
	Mechanical ventilation	104 (50)	32 (44)	0.38
	Corticosteroid treatment	148 (72)	57 (79)	0.22
**Chest CT scan**		N=212	N=66	
	Age ≥ 65	71 (34)	22 (33)	0.98
	Male	151 (71)	52 (79)	0.23
	Diabetes	46 (22)	14 (21)	0.93
	Hypertension/ cardiovascular disease	104 (49)	35 (53)	0.57
	Chronic lung disease	49 (23)	10 (15)	0.17
	Mechanical ventilation	107 (50)	29 (44)	0.35
	Corticosteroid treatment	155 (73)	50 (76)	0.67

For peer review only

Supplementary Table 4. Univariate analysis of patient- and clinical characteristics in relation to health-related quality of life (HRQL) in COVID-19 intensive care unit survivors presented as adjusted mean score differences (MSD) with 95% confidence intervals (CI).

**Variables**	Number	**Physical function**<br>MSD (95%CI)<br>p-value	**Role physical**<br>MSD (95%CI)<br>p-value	**Bodily pain**<br>MSD (95%CI)<br>p-value	**General health**<br>MSD (95%CI)<br>p-value	**Vitality**<br>MSD (95%CI)<br>p-value	**Social function**<br>MSD (95%CI)<br>p-value	**Role emotional**<br>MSD (95%CI)<br>p-value	**Mental health**<br>MSD (95%CI)<br>p-value
**Age, years**									
≤65	120	Reference 1.0	Reference 1.0	Reference 1.0	Reference 1.0	Reference 1.0	Reference 1.0	Reference 1.0	Reference 1.0
>65	66	-13.3*<br>(-21.3 to -5.3)<br><0.01	1.0<br>(-12.5 to 14.4)<br>0.89	-5.2<br>(-14.1 to 3.6)<br>0.24	-1.1<br>(-7.9 to 5.6)<br>0.74	3.3<br>(-4.2 to 10.8)<br>0.38	-1.7<br>(-10.4 to 7.0)<br>0.70	3.4<br>(-9.6 to 16.3)<br>0.61	2.5<br>(-3.7 to 8.8)<br>0.42
**Sex**									
Female	53	Reference 1.0	Reference 1.0	Reference 1.0	Reference 1.0	Reference 1.0	Reference 1.0	Reference 1.0	Reference 1.0
Male	133	6.5<br>(-2.2 to 15.2)<br>0.14	13.8<br>(-0.3 to 27.9)<br>0.06	4.6<br>(-4.7 to 14.0)<br>0.33	5.6<br>(-1.6 to 12.7)<br>0.12	8.6*<br>(0.7-16.4)<br>0.03	10.2*<br>(1.1 to 19.2)<br>0.03	1.8<br>(-11.9 to 15.5)<br>0.80	1.8<br>(-4.8 to 8.4)<br>0.60
**Diabetes**									
No	147	Reference 1.0	Reference 1.0	Reference 1.0	Reference 1.0	Reference 1.0	Reference 1.0	Reference 1.0	Reference 1.0
Yes	39	-11.5*<br>(-21.0 to -2.0)<br>0.02	-6.74<br>(-22.5 to 9.0)<br>0.4	-10.2<br>(-20.5 to 0.1)<br>0.05	-10.0*<br>(-17.8 to -2.2)<br>0.01	-4.5<br>(-13.3 to 4.3)<br>0.31	-3.1<br>(-13.3 to 7.1)<br>0.55	-10.1<br>(-25.2 to 5.1)<br>0.19	-8.3*<br>(-15.6 to -1.1)<br>0.02
**Hypertension/ cardiovascular disease**									
No	87	Reference 1.0	Reference 1.0	Reference 1.0	Reference 1.0	Reference 1.0	Reference 1.0	Reference 1.0	Reference 1.0
Yes	99	-7.9*<br>(-15.7 to -0.7)<br>0.05	0.5<br>(-12.4 to 13.4)<br>0.94	-7.4<br>(-15.8 to 1.0)<br>0.08	0.0<br>(-6.5 to 6.5)<br>1.0	2.3<br>(-4.9 to 9.5)<br>0.52	1.2<br>(-7.1 to 9.5)<br>0.78	0.8<br>(-11.6 to 13.2)<br>0.90	1.95<br>(-4.1 to 7.9)<br>0.52
**Chronic lung disease**									
No	144	Reference 1.0	Reference 1.0	Reference 1.0	Reference 1.0	Reference 1.0	Reference 1.0	Reference 1.0	Reference 1.0
Yes	42	-7.0<br>(-16.3 to 2.4)<br>0.14	-15.1<br>(-30.2 to -0.0)<br>0.05	-5.4<br>(-15.5 to 4.6)<br>0.29	-7.2<br>(-14.8 to 0.4)<br>0.06	-9.7*<br>(-18.1 to -1.3)<br>0.02	-6.0<br>(-15.8 to 3.9)<br>0.23	-20.5*<br>(-34.8 to -6.1)<br><0.01	-2.6<br>(-9.7 to 4.4)<br>0.46

For peer review only

**Ventilation support**<br>HFNO/NIV<br>invasive ventilation	 <br>95<br>91	 <br>Reference 1.0<br>-5.6<br>(-13.4 to 2.3)<br>0.16	 <br>Reference 1.0<br>-12.4<br>(-25.1 to 0.4)<br>0.06	 <br>Reference 1.0<br>-12.3*<br>(-20.6 to -4.0)<br><0.01	 <br>Reference 1.0<br>-2.2<br>(-8.6 to 4.3)<br>0.5	 <br>Reference 1.0<br>-1.4<br>(-8.6 to 5.8)<br>0.70	 <br>Reference 1.0<br>-7.8<br>(-16.0 to 0.5)<br>0.06	 <br>Reference 1.0<br>-1.26<br>(-13.7 to 11.2)<br>0.84	 <br>Reference 1.0<br>2.8<br>(-3.2 to 8.8)<br>0.36
**In-ICU corticosteroids**<br>No<br>Yes	 <br>43<br>143	 <br>Reference 1.0<br>-3.6<br>(-12.9 to 5.7)<br>0.44	 <br>Reference 1.0<br>5.7<br>(-9.4 to 20.8)<br>0.46	 <br>Reference 1.0<br>3.3<br>(-6.6 to 13.3)<br>0.5	 <br>Reference 1.0<br>1.9<br>(-5.7 to 9.5)<br>0.6	 <br>Reference 1.0<br>3.6<br>(-4.8 to 12.0)<br>0.40	 <br>Reference 1.0<br>2.6<br>(-7.2 to 12.4)<br>0.60	 <br>Reference 1.0<br>9.1<br>(-5.4 to 23.6)<br>0.22	 <br>Reference 1.0<br>6.4<br>(-0.6 to 13.3)<br>0.07

Abbreviations: HFNO=high flow nasal oxygen; NIV=non-invasive ventilation. *Statistically significant p-value <0.05.

For peer review only

STROBE (Strengthening The Reporting of OBservational Studies in Epidemiology) Checklist

A checklist of items that should be included in reports of observational studies. You must report the page number in your manuscript where you consider each of the items listed in this checklist. If you have not included this information, either revise your manuscript accordingly before submitting or note N/A.

Note: An Explanation and Elaboration article discusses each checklist item and gives methodological background and published examples of transparent reporting. The STROBE checklist is best used in conjunction with this article (freely available on the Web sites of PLoS Medicine at http://www.plosmedicine.org/, Annals of Internal Medicine at http://www.annals.org/, and Epidemiology at http://www.epidem.com/). Information on the STROBE Initiative is available at www.strobe-statement.org.

Section and Item	Item No.	Recommendation	Reported on Page No.
**Title and Abstract**	1	(*a*) Indicate the study's design with a commonly used term in the title or the abstract	
		(*b*) Provide in the abstract an informative and balanced summary of what was done and what was found	
**Introduction**			
Background/Rationale	2	Explain the scientific background and rationale for the investigation being reported	
Objectives	3	State specific objectives, including any prespecified hypotheses	
**Methods**			
Study Design	4	Present key elements of study design early in the paper	
Setting	5	Describe the setting, locations, and relevant dates, including periods of recruitment, exposure, follow-up, and data collection	
Participants	6	(*a*) *Cohort study*—Give the eligibility criteria, and the sources and methods of selection of participants. Describe methods of follow-up<br>*Case-control study*—Give the eligibility criteria, and the sources and methods of case ascertainment and control selection. Give the rationale for the choice of cases and controls<br>*Cross-sectional study*—Give the eligibility criteria, and the sources and methods of selection of participants	
		(*b*) *Cohort study*—For matched studies, give matching criteria and number of exposed and unexposed<br>*Case-control study*—For matched studies, give matching criteria and the number of controls per case	
Variables	7	Clearly define all outcomes, exposures, predictors, potential confounders, and effect modifiers. Give diagnostic criteria, if applicable	

For peer review only

Section and Item	Item No.	Recommendation	Reported on Page No.
Data Sources/ Measurement	8*	For each variable of interest, give sources of data and details of methods of assessment (measurement). Describe comparability of assessment methods if there is more than one group	
Bias	9	Describe any efforts to address potential sources of bias	
Study Size	10	Explain how the study size was arrived at	
Quantitative Variables	11	Explain how quantitative variables were handled in the analyses. If applicable, describe which groupings were chosen and why	
Statistical Methods	12	(*a*) Describe all statistical methods, including those used to control for confounding	
		(*b*) Describe any methods used to examine subgroups and interactions	
		(*c*) Explain how missing data were addressed	
		(*d*) *Cohort study*—If applicable, explain how loss to follow-up was addressed<br>*Case-control study*—If applicable, explain how matching of cases and controls was addressed<br>*Cross-sectional study*—If applicable, describe analytical methods taking account of sampling strategy	
		(*e*) Describe any sensitivity analyses	
**Results**			
Participants	13*	(a) Report numbers of individuals at each stage of study—eg numbers potentially eligible, examined for eligibility, confirmed eligible, included in the study, completing follow-up, and analysed	
		(b) Give reasons for non-participation at each stage	
		(c) Consider use of a flow diagram	
Descriptive Data	14*	(a) Give characteristics of study participants (eg demographic, clinical, social) and information on exposures and potential confounders	
		(b) Indicate number of participants with missing data for each variable of interest	
		(c) *Cohort study*—Summarise follow-up time (eg, average and total amount)	
Outcome Data	15*	*Cohort study*—Report numbers of outcome events or summary measures over time	
		Case-control study—Report numbers in each exposure category, or summary measures of exposure	
		Cross-sectional study—Report numbers of outcome events or summary measures	

For peer review only

Section and Item	Item No.	Recommendation	Reported on Page No.
Main Results	16	(*a*) Give unadjusted estimates and, if applicable, confounder-adjusted estimates and their precision (eg, 95% confidence interval). Make clear which confounders were adjusted for and why they were included	
		(*b*) Report category boundaries when continuous variables were categorized	
		(*c*) If relevant, consider translating estimates of relative risk into absolute risk for a meaningful time period	
Other Analyses	17	Report other analyses done—eg analyses of subgroups and interactions, and sensitivity analyses	
**Discussion**			
Key Results	18	Summarise key results with reference to study objectives	
Limitations	19	Discuss limitations of the study, taking into account sources of potential bias or imprecision. Discuss both direction and magnitude of any potential bias	
Interpretation	20	Give a cautious overall interpretation of results considering objectives, limitations, multiplicity of analyses, results from similar studies, and other relevant evidence	
Generalisability	21	Discuss the generalisability (external validity) of the study results	
**Other Information**			
Funding	22	Give the source of funding and the role of the funders for the present study and, if applicable, for the original study on which the present article is based	

*Give information separately for cases and controls in case-control studies and, if applicable, for exposed and unexposed groups in cohort and cross-sectional studies.

Once you have completed this checklist, please save a copy and upload it as part of your submission. DO NOT include this checklist as part of the main manuscript document. It must be uploaded as a separate file.

For peer review only
